# Electron‐Rich Nickel Enabled by Charge Transfer in Multimetal Nanostructures for Efficient Ammonia‐to‐Hydrogen Conversion

**DOI:** 10.1002/advs.78184

**Published:** 2026-10-06

**Authors:** Donghyeok Kim, Su‐Jin Kim, Gayeong Kim, Hyoseok Kim, Jongkyoung Kim, Myeung‐Jin Lee, Bora Jeong, Minkyu Kim, Hong‐Dae Kim, Seungho Cho

**Affiliations:** ^1^ Department of Materials Science and Engineering Ulsan National Institute of Science and Technology (UNIST) Ulsan Republic of Korea; ^2^ Ulsan Technology Application Division Korea Institute of Industrial Technology (KITECH) Ulsan Republic of Korea; ^3^ Department of Chemical Engineering Yeungnam University Gyeongsan Republic of Korea; ^4^ UNIST InnoCORE hydro*studio Ulsan National Institute of Science and Technology (UNIST) Ulsan Republic of Korea; ^5^ Graduate School of Semiconductor Materials and Devices Engineering Ulsan National Institute of Science and Technology (UNIST) Ulsan Republic of Korea

**Keywords:** ammonia decomposition, charge transfer, hydrogen production, LDH‐derived NiCrAl catalysts, nickel active sites

## Abstract

Achieving noble‐metal‐like reactivity in earth‐abundant catalysts requires materials platforms that can simultaneously stabilize active sites and program their electronic states. Ammonia‐to‐hydrogen conversion is a demanding example, because Ru‐free catalysts must overcome the intrinsically sluggish kinetics of ammonia decomposition. Here, we report a catalyst design that couples precursor‐level cation mixing and active‐site dispersion with charge‐transfer engineering of Ni to address this challenge. Density functional theory calculations identify Cr as the most effective electron‐donating promoter for Ni among metal cations incorporable into the precursor framework. Guided by this prediction, we construct a NiCrAl reduced mixed‐metal oxide catalyst in which atomically mixed precursors stabilize highly dispersed Ni species, while Cr‐to‐Ni charge transfer generates electron‐rich Ni active sites. Multiscale spectroscopic analyses verify this electronic modulation, and combined theoretical and in situ mechanistic analyses indicate that electron‐rich Ni favors NH_3_ adsorption in a reaction‐relevant Lewis‐acid configuration, favorably modifies the dehydrogenation energetics, and weakens N_2_ binding to facilitate product desorption. NiCrAl‐RMMO reaches 95.5% NH_3_ conversion at 550°C and 22.83 mmol H_2_ g_cat_^−1^ min^−1^ at 500°C, with stable operation over 100 h. More broadly, this work establishes charge‐transfer engineering in LDH‐derived mixed‐metal nanostructures as a general strategy for designing high‐performance, Ru‐free catalysts for ammonia‐to‐hydrogen conversion.

## Graphical abstract

A charge‐transfer‐engineered Ni catalyst derived from an atomically mixed layered precursor enables efficient ammonia‐to‐hydrogen conversion. Cr‐to‐Ni electron transfer creates electron‐rich Ni active sites that favor NH_3_ adsorption, modify the dehydrogenation energetics, and weaken N_2_ binding to facilitate product desorption. This electronically tuned catalyst delivers high hydrogen productivity and durable catalytic performance.

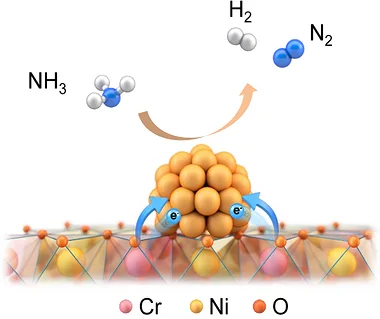

## 1 Introduction

The transition toward a hydrogen‐based energy economy has intensified the search for practical routes to produce, transport, and utilize H_2_ with minimal carbon emissions [1, 2]. At the same time, it has brought a broader materials challenge into sharp focus: how to design earth‐abundant catalysts that can approach noble‐metal‐like reactivity through simultaneous control of active‐site dispersion and electronic structure. Although water electrolysis is widely regarded as the most desirable route for green hydrogen production, its large‐scale implementation remains constrained by high production costs and the difficulty of long‐distance H_2_ transport and storage. Because molecular hydrogen has a low volumetric energy density, direct overseas import and distribution require substantial investments in liquefaction, compression, and dedicated infrastructure. In this context, ammonia (NH_3_) has emerged as one of the most attractive hydrogen carriers because it is carbon‐free, possesses a high gravimetric hydrogen density, can be liquefied under relatively mild conditions, and is already supported by established global transportation and storage networks [3, 4, 5, 6]. These advantages make NH_3_ a compelling platform for building an economically viable hydrogen supply chain. However, the practicality of this platform ultimately depends on the efficiency of ammonia cracking, because NH_3_ decomposition is strongly endothermic and represents one of the most energy‐intensive and costly steps in the overall process [7]. Therefore, the development of highly active and durable catalysts for NH_3_ decomposition is a central requirement for realizing cost‐effective hydrogen production from ammonia.

Among the catalysts investigated to date, Ru‐based systems exhibit the highest activity for NH_3_ decomposition [8, 9, 10, 11, 12]. Nevertheless, the scarcity and high cost of Ru pose serious barriers to widespread deployment, particularly for large‐scale hydrogen production. This limitation has motivated extensive efforts to develop catalysts based on earth‐abundant transition metals [13, 14, 15, 16, 17, 18]. Among them, Ni is particularly attractive because it combines relatively high intrinsic activity with low cost, making it one of the most promising candidates for practical Ru‐free NH_3_ decomposition [19]. Despite this potential, however, Ni‐based catalysts still suffer from a fundamental limitation: they generally require substantially higher operating temperatures than Ru‐based catalysts because of less favorable reaction energetics [20, 21]. More importantly, the catalytic performance of Ni is governed not only by the number and dispersion of exposed active sites, but also by the electronic structure of those sites, which dictates the adsorption, activation, transformation, and desorption of key reaction intermediates [22, 23]. Thus, further advances in Ni catalysis require more than conventional dispersion control; they demand a catalyst design strategy that can simultaneously stabilize highly dispersed Ni species and deliberately tune their redox and electronic states to overcome the intrinsic kinetic bottlenecks of NH_3_ decomposition.

Layered double hydroxides (LDHs) provide a uniquely suitable platform for such a strategy [24, 25]. Owing to their atomic‐level cation mixing within brucite‐like layers, multicomponent LDHs enable exceptionally uniform metal dispersion and precise compositional control [26]. Upon thermal transformation, LDH precursors generate mixed‐metal oxides (MMOs) in which catalytically active metals can remain highly dispersed, thereby suppressing aggregation and sintering under harsh reaction conditions [27]. This structural advantage is especially important for Ni‐based catalysts operating at elevated temperatures. However, geometric stabilization alone is insufficient to unlock the full catalytic potential of Ni. Even when Ni is nanosized and well dispersed, its intrinsic activity remains strongly limited by the energetics of elementary reaction steps, particularly those associated with N─H bond cleavage and the removal of strongly adsorbed nitrogen species. Accordingly, a more effective catalyst platform must integrate structural stabilization with purposeful electronic modulation of the Ni active sites [28].

Here, we report a rational catalyst design that addresses these two requirements simultaneously by using a NiCrAl LDH precursor as a programmable platform for both atomic dispersion and redox‐state engineering of Ni. In this design, Al serves as a structurally robust matrix that helps generate a high‐surface‐area support, while Cr is introduced as an electronic promoter selected through density functional theory (DFT) calculations for its strong ability to donate electron density to Ni. The resulting electronic interaction between Cr and Ni produces electron‐enriched Ni sites after reduction [28, 29, 30]. This electronically tuned Ni state is expected to facilitate NH_3_ activation by promoting back‐donation into antibonding orbitals, while also weakening the adsorption strength of nitrogen‐containing intermediates and thereby lowering the barrier for nitrogen removal [31, 32]. Because the rate‐limiting step in ammonia decomposition is widely associated with N─N recombination and subsequent N_2_ desorption on non‐noble metal surfaces, such modulation of the Ni redox state provides a direct route to reducing the intrinsic kinetic barrier of the reaction rather than merely increasing the number of accessible sites.

Comprehensive experimental and theoretical analyses support this mechanism. X‐ray absorption near‐edge structure (XANES) measurements and DFT calculations reveal electron transfer from Cr to Ni, while in situ X‐ray diffraction and NH_3_ temperature‐programmed reduction verify the enhanced reducibility and dynamic structural evolution of the catalyst. As a result, the optimized reduced mixed‐metal oxide derived from NiCrAl LDH exhibits outstanding NH_3_ conversion and H_2_ production rates, surpassing conventional non‐noble Ni‐based catalysts while maintaining stable activity over 100 h of continuous operation. More importantly, this work demonstrates that promoter‐induced control over the Ni redox state can directly regulate the key reaction energetics of ammonia decomposition. Beyond the specific NiCrAl system, these findings establish a general materials design principle in which atomically mixed layered precursors are leveraged to couple geometric stabilization with electronic‐state engineering, offering a scalable strategy for the development of high‐performance, Ru‐free catalysts for hydrogen generation from ammonia.

## 2 Results and Discussion

### 2.1 Computational Design and Structural Characterization of NiCrAl LDH‐Derived Mixed‐Metal Nanostructures

We began by computationally screening metal cations compatible with the LDH lattice to identify a promoter that could most effectively enrich the electron density of Ni (Figure 1a). Among the candidate elements examined, Cr showed the strongest electron‐donating effect toward Ni, immediately identifying it as the most promising choice for tuning the redox state of Ni within the LDH‐derived catalyst platform. Guided by this result, we then focused on Cr‐containing systems to elucidate the underlying electronic interactions in greater detail.

**FIGURE 1:**
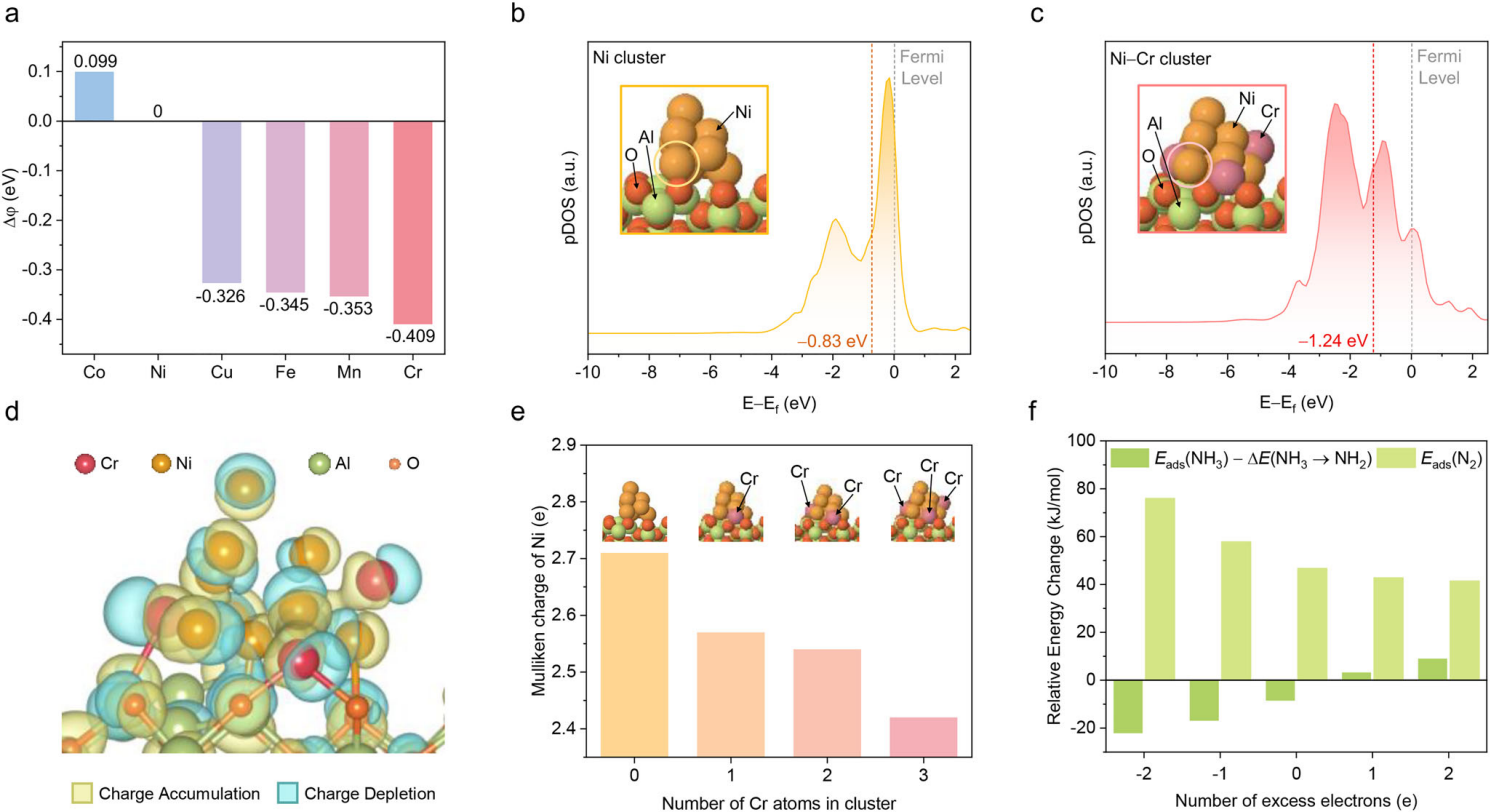
Computational screening and descriptor analysis for electronically tuned LDH‐derived Ni‐based nanostructures. (a) Work‐function‐based screening of transition‐metal dopants relative to Ni, expressed as Δφ = φ(M) − φ(Ni). (b,c) pDOS of Ni in the (b) Ni cluster and (c) Ni–Cr cluster. (d) Charge‐density‐difference (CDD) map of Ni/Al_2_O_3_ containing interfacial Cr. (e) Charge accumulation on supported Ni clusters as a function of interfacial Cr content. (f) Dependence of the NH_3_‐activation descriptor, $E_{\mathrm{ads}}\left(NH_{3}\right)-\Delta E\left(NH_{3}\to NH_{2}\right)$ and the N_2_ adsorption energy as a function of the imposed electron count on Ni(111).

Projected density of states (pDOS) analysis revealed that Cr incorporation induces a distinct downward shift in the Ni d‐band center (Figure 1b,c), reflecting the increased filling of the Ni d‐band and thus a more electron‐rich Ni state [28, 33]. Charge density difference analysis provided direct real‐space evidence for this electronic redistribution (Figure 1d): electron depletion was localized around Cr, whereas electron accumulation was concentrated around Ni, unambiguously confirming charge transfer from Cr to Ni. These findings establish Cr as an effective electronic promoter that modifies the local electronic structure of Ni rather than serving as a passive secondary component.

Moreover, this electronic interaction was not binary but composition‐dependent. Increasing the number of Cr atoms progressively amplified the electron accumulation around Ni, demonstrating that the Ni electronic state can be systematically regulated by the Cr content (Figure 1e). Notably, the same compositional trend was reproduced in simplified Ni–Cr cluster calculations without Al (Figure S1), confirming that the essential origin of the electronic modulation lies in the direct Ni–Cr interaction itself. Importantly, additional DFT calculations using Ni_10_ clusters supported on NiAl_2_O_4_ and NiCr_2_O_4_ spinel surfaces further confirmed that Cr incorporation consistently enriches the electron density of Ni even in catalyst‐relevant spinel environments (Figure S2).

To determine whether the electron‐enriched Ni state is genuinely beneficial for ammonia decomposition, we next examined two representative reaction descriptors: the NH_3_ activation descriptor, defined as  $E_{\mathrm{ads}}\left(NH_{3}\right)-\Delta E\left(NH_{3}\to NH_{2}\right)$, and the N_2_ adsorption energy, *E*_ads_(N_2_), which captures the ease of product desorption (Figure 1f and Figure S3). A clear trend emerged as the electron density of Ni increased. Here, the electron enrichment or depletion was implemented by systematically varying the total number of electrons in the Ni(111) slab using the NELECT parameter in VASP, with a compensating homogeneous background charge introduced for the resulting charged periodic cell. Under electron‐deficient conditions, the NH_3_ activation descriptor remained negative, indicating unfavorable NH_3_ activation. In contrast, electron enrichment shifted this descriptor to positive values, revealing that NH_3_ activation becomes progressively more favorable on electron‐rich Ni sites. Simultaneously, *E*_ads_ (N_2_) decreased with increasing electron density, indicating weaker N_2_ binding and thus easier product desorption. Taken together, these results show that electron enrichment of Ni beneficially modulates two critical energetic requirements of ammonia decomposition at once: it facilitates the activation of NH_3_ while also promoting the release of N_2_. Accordingly, the electron‐rich regime offers the most favorable energetic window for efficient ammonia decomposition among the conditions examined [34].

Based on the computational identification of Cr as an effective electron‐donating promoter for Ni, we next synthesized a series of LDH‐derived mixed‐metal nanostructures to experimentally realize this electronically tuned catalyst platform. The overall synthetic route is illustrated in Figure 2a. Briefly, an LDH precursor was first prepared, followed by calcination in air to generate the mixed‐metal oxide (MMO) intermediate, and subsequent reduction under pure NH_3_ to produce the final reduced mixed‐metal oxide (RMMO) nanostructure. The metal compositions of the synthesized samples are summarized in Table S1.

**FIGURE 2:**
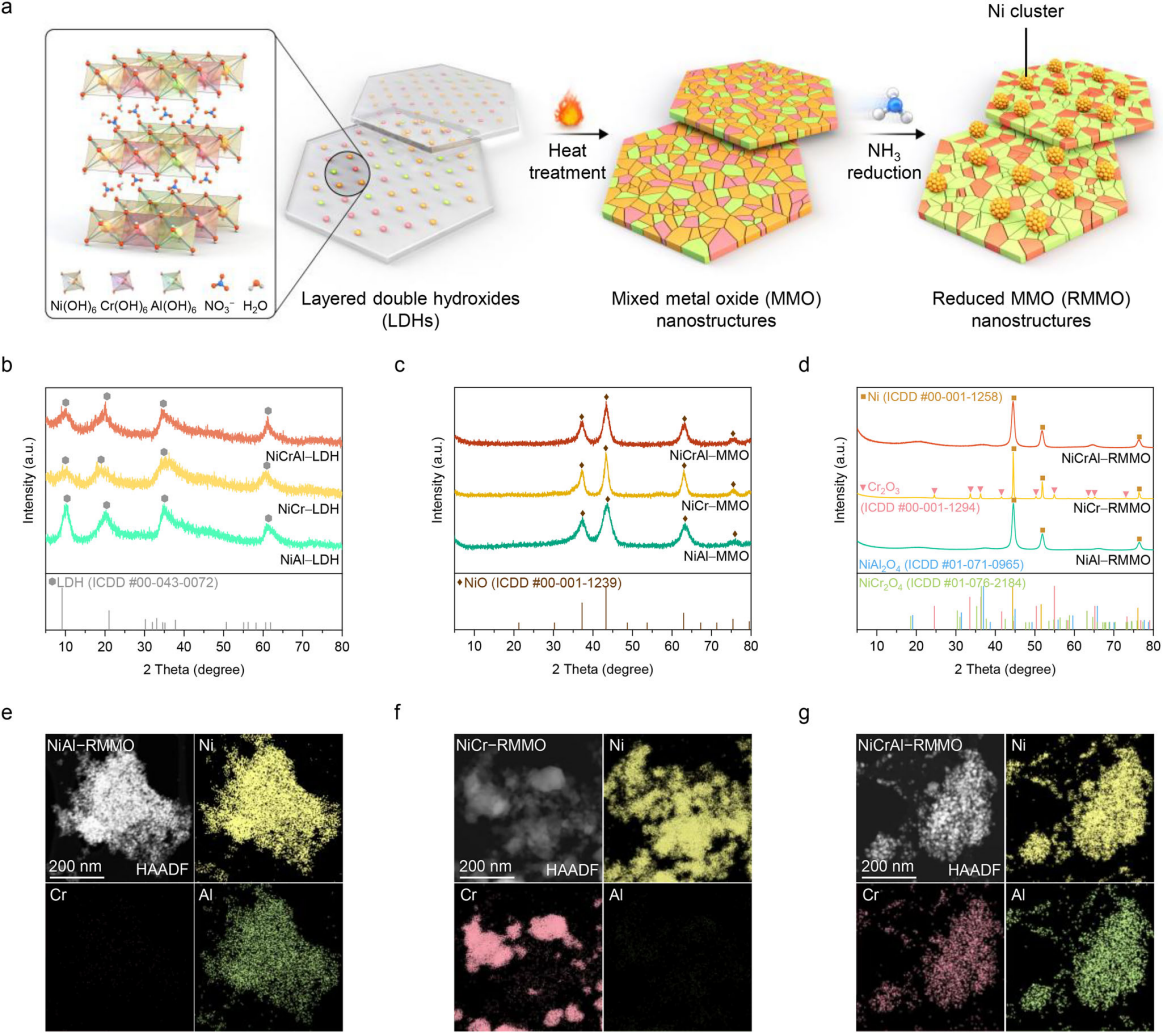
Structural evolution of LDH‐derived mixed‐metal nanostructures and nanoscale elemental distributions of the reduced products. (a) Schematic illustration of the synthesis of the LDH, MMO, and RMMO nanostructures. (b–d) XRD patterns showing the phase evolution from the (b) LDH precursor to the (c) MMO intermediate and the (d) RMMO nanostructure. (e–g) HAADF‐STEM images and corresponding EDS elemental maps of the (e) NiAl‐RMMO, (f) NiCr‐RMMO, and (g) NiCrAl‐RMMO nanostructures.

The phase evolution during the synthesis was examined by X‐ray diffraction (XRD) (Figure 2b–d). The precursor exhibited diffraction peaks characteristic of a hydrotalcite‐like LDH structure containing interlayer NO_3_^−^ anions (ICDD #00‐043‐0072), confirming the successful formation of the layered precursor. After calcination in air at 600°C for 2 h, the LDH was transformed into the corresponding MMO nanostructure, whose diffraction pattern was dominated by reflections assignable to NiO (ICDD #00‐001‐1239). Subsequent reduction under pure NH_3_ converted the NiO phase into metallic Ni in all samples (ICDD #00‐001‐1258). However, the resulting phase compositions and structural features strongly depended on the accompanying support components. In the Al‐free NiCr‐RMMO sample, distinct and sharp diffraction peaks corresponding to Cr_2_O_3_ (ICDD #00‐001‐1294) were observed in addition to metallic Ni, indicating pronounced phase separation between Ni and Cr. By contrast, NiAl‐RMMO and NiCrAl‐RMMO, both of which contain Al as a support component, showed substantially broader Ni diffraction peaks, suggesting that the Ni species were more finely dispersed. Furthermore, both NiAl‐RMMO and NiCrAl‐RMMO contained coexisting amorphous alumina and spinel phases (Figure S4), with NiAl_2_O_4_ formed in NiAl‐RMMO and NiCr_2_O_4_ identified in NiCrAl‐RMMO.

The nanostructural characteristics of the RMMO samples were further examined by Cs‐corrected scanning transmission electron microscopy (STEM) (Figure S5). High‐angle annular dark‐field STEM (HAADF‐STEM) images and the corresponding EDS elemental maps are shown in Figure 2e–g. Consistent with the XRD results, NiCr‐RMMO without Al exhibited severe aggregation of Ni‐ and Cr‐containing domains together with clear phase separation between the two components. In sharp contrast, both NiAl‐RMMO and NiCrAl‐RMMO displayed a homogeneous spatial distribution of their constituent elements throughout the nanostructures, indicating that Al plays a critical role in suppressing phase segregation and stabilizing highly dispersed mixed‐metal architectures [35]. This structural difference was also reflected in the specific surface areas of the samples (Table S2). Notably, uniform elemental distributions were already observed in both the LDH precursors and MMO intermediates (Figures S6 and S7), suggesting that the compositional homogeneity established in the LDH precursor is preserved to a substantial extent during the subsequent phase transformations. Additional scanning electron microscopy (SEM) images of all synthesized samples are provided in Figures S8–S10.

### 2.2 Electron Transfer from Cr to Ni in MMO and RMMO Nanostructures

DFT calculations predicted that Cr incorporation would enrich the electron density of Ni active sites, and the preceding structural analyses confirmed the successful formation of the corresponding LDH‐derived nanostructures. We next sought to experimentally verify whether such charge transfer indeed occurs in the synthesized MMO and RMMO and to determine how it evolves with composition.

To probe the electronic structure of Ni, we first performed X‐ray absorption fine structure (XAFS) analysis on the MMO and RMMO nanostructures. In the Ni K‐edge XANES spectra of the MMO samples (Figure 3a), the Cr‐containing compositions showed lower white‐line intensities than the Cr‐free sample, suggesting a modification of the local electronic environment of Ni toward a more metallic‐like character. The corresponding k^2^‐weighted Fourier‐transform EXAFS spectra of the MMO nanostructures are provided in Figure S11. This result suggests that Cr‐induced electronic modulation of Ni is already established at the MMO stage, prior to the final reduction step. The same trend became even more pronounced in the RMMO catalysts used for ammonia decomposition (Figure 3b). Specifically, the white‐line intensity decreased in the order of NiAl‐RMMO, NiCrAl‐RMMO, and NiCr‐RMMO, reflecting composition‐dependent differences in the local electronic environment of Ni [36]. This interpretation is further supported by the EXAFS results for the RMMO nanostructures, in which the Ni─Ni coordination environment of NiCr‐RMMO was found to be most similar to that of Ni foil (Figure 3c and Figure S12) [37, 38, 39]. Together, the XANES and EXAFS analyses consistently indicate that Cr incorporation drives Ni toward a more electron‐rich state.

**FIGURE 3:**
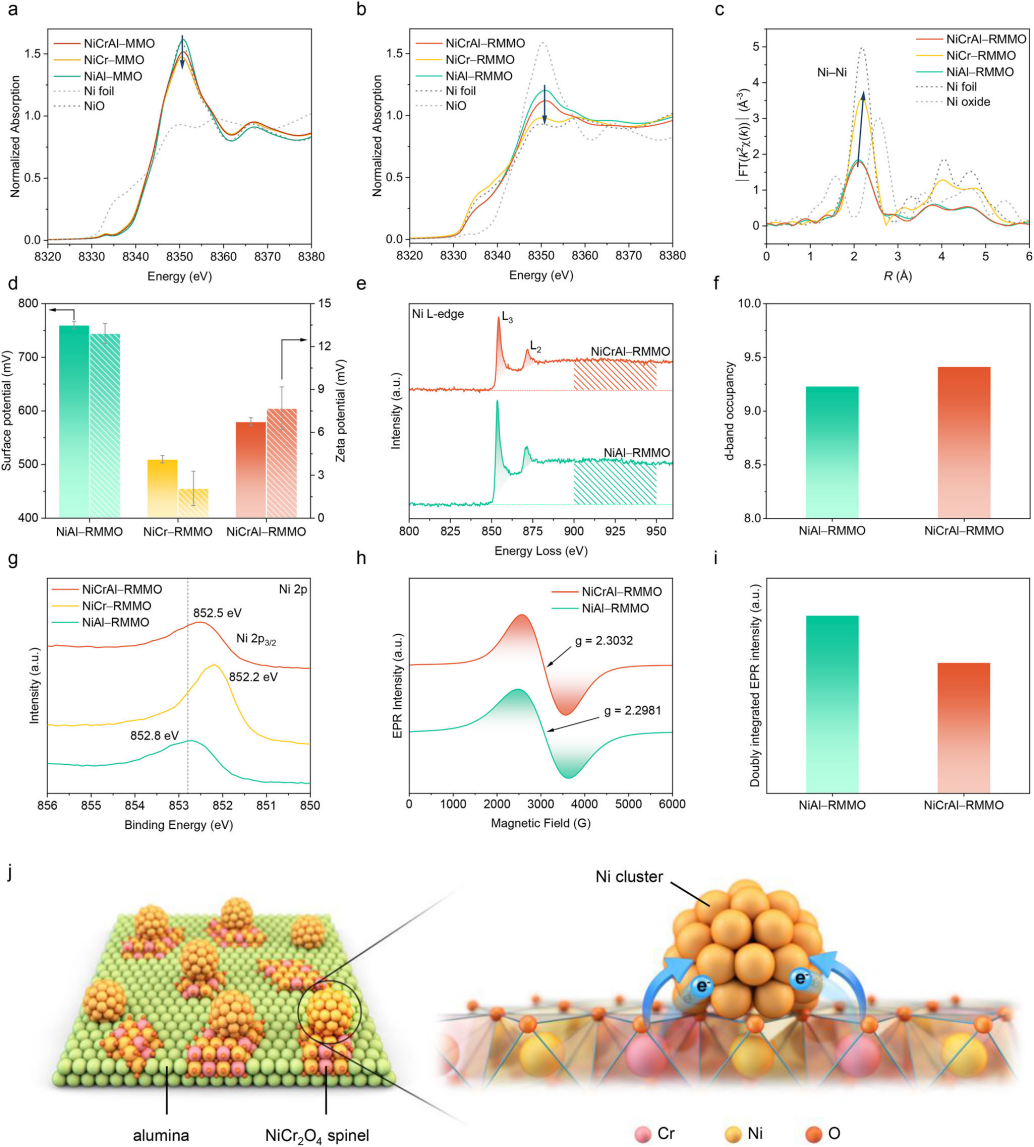
Multiscale electronic‐structure analyses revealing electron transfer from Cr to Ni in LDH‐derived RMMO nanostructures. (a) Ni K‐edge XANES spectra of the MMO nanostructures. (b) Ni K‐edge XANES spectra and (c) *k* ^2^‐weighted Fourier‐transform EXAFS spectra of the RMMO nanostructures. (d) Surface potential profiles obtained from the KPFM line scans shown in Figure S13, together with the zeta potentials of the RMMO nanostructures. (e) Ni L_2,3_‐edge ELNES spectra and (f) the corresponding d‐band occupancies of NiAl‐RMMO and NiCrAl‐RMMO. (g) Depth‐profiled Ni 2p XPS spectra of the RMMO nanostructures. (h) EPR spectra and (i) doubly integrated EPR intensities of NiAl‐RMMO and NiCrAl‐RMMO. (j) Schematic illustration of electron transfer from Cr‐containing domains to adjacent Ni sites in the NiCrAl‐RMMO nanostructure.

The surface electronic characteristics of the RMMO nanostructures were further examined by Kelvin probe force microscopy (KPFM) and zeta potential measurements (Figure 3d and Figure S13). Both techniques revealed the same composition‐dependent trend, with the electron‐rich character of the surface increasing in the order of NiAl‐RMMO, NiCrAl‐RMMO, and NiCr‐RMMO. The agreement between these surface‐sensitive measurements and the XAFS results indicates that Cr‐induced electron enrichment is not limited to the bulk coordination environment but is also reflected in the surface regions that are directly relevant to catalysis.

To directly assess changes in the occupancy of the Ni 3d states, we additionally performed STEM‐electron energy loss spectroscopy (EELS) analysis (Figure 3e,f and Figure S14). The electron‐loss near‐edge structure (ELNES) spectra in the Ni L_2,3_‐edge region displayed characteristic white‐line features associated with transitions to unoccupied 3d states in all RMMO samples. Because the absolute white‐line intensity is influenced by factors such as sample thickness, the spectra were normalized using the ratio to the integrated post‐edge background [40]. On this basis, NiCrAl‐RMMO exhibited a higher d‐band occupancy than NiAl‐RMMO, indicating that the Ni 3d orbitals become more electron‐filled upon Cr incorporation [28]. This result provides direct local‐scale evidence that Cr modifies the electronic structure of Ni by increasing its electron occupancy.

We then used depth‐profiled X‐ray photoelectron spectroscopy (XPS) to identify the metallic Ni species beneath the surface oxide layer of the RMMO nanostructures (Figure 3g and Figure S15). Before etching, the surface spectra were dominated by NiO‐related features, whereas after 30 s of etching, the Ni° component became clearly visible (Figures S16–S21). Importantly, the binding energy of the metallic Ni 2p_3/2_ peak systematically decreased from 852.8 eV for NiAl‐RMMO to 852.5 eV for NiCrAl‐RMMO and 852.2 eV for NiCr‐RMMO. These values indicate composition‐dependent differences in the electronic state of metallic Ni, while the pronounced structural differences of NiCr‐RMMO preclude assigning this three‐sample trend solely to Cr content [41, 42, 43]. Because Ar^+^ sputtering can induce partial reduction of Ni species, the depth‐profiled XPS results were evaluated across matched etching times. Although a minor contribution from sputter‐induced reduction cannot be completely excluded, the relative Ni 2p_3/2_ binding‐energy difference between NiAl‐RMMO and NiCrAl‐RMMO was maintained at 30, 60, and 120 s of etching (Figure S17), supporting the reproducibility of the relative shift with respect to sputtering duration. To further examine the composition‐dependent electronic modulation within the NiCrAl system, XPS analysis was performed on catalysts with systematically varied Cr contents (Figure S22). All Cr‐containing NiCrAl‐RMMO compositions exhibited lower Ni binding energies than NiAl‐RMMO, while the differences among the three Cr‐containing compositions were relatively modest, indicating Cr‐induced electronic modulation without a strong monotonic Cr‐content dependence within the investigated composition range. A similar tendency was also captured by electron paramagnetic resonance (EPR) spectroscopy (Figure 3h,i), where NiCrAl‐RMMO showed a smaller signal area than NiAl‐RMMO, consistent with a reduced population of unpaired electron‐related species in the more electron‐rich Ni environment [28, 44, 45].

Taken together, these complementary bulk‐, surface‐, and local‐scale characterizations consistently verify electron transfer from Cr to Ni in the LDH‐derived nanostructures. Cr therefore functions as an effective electronic promoter that modifies the redox state and electron occupancy of adjacent Ni species. As schematically illustrated in Figure 3j, electron donation from Cr‐containing support domains to Ni clusters generates electron‐rich Ni active sites, providing the electronic environment required to facilitate ammonia decomposition.

### 2.3 Charge‐Transfer‐Induced Modulation of the Reducibility of MMO Nanostructures

We next examined whether the Cr‐to‐Ni electron transfer identified above translates into a measurable change in the reducibility of the MMO nanostructures. For this purpose, in situ XRD measurements were conducted under H_2_, while the phase evolution from LDH to MMO was separately tracked under air (Figure 4a–c and Figure S23). All MMO samples underwent gradual reduction of NiO to metallic Ni with increasing temperature, but the onset of this transformation was strongly composition‐dependent. The reduction began at around 650°C for NiAl‐MMO, 600°C for NiCrAl‐MMO, and 550°C for NiCr‐MMO, clearly showing that Cr incorporation lowers the reduction temperature. Combined with the spectroscopic evidence presented above, this behavior indicates that electron donation from Cr renders Ni more electron‐rich and thereby facilitates its reduction [46, 47].

**FIGURE 4:**
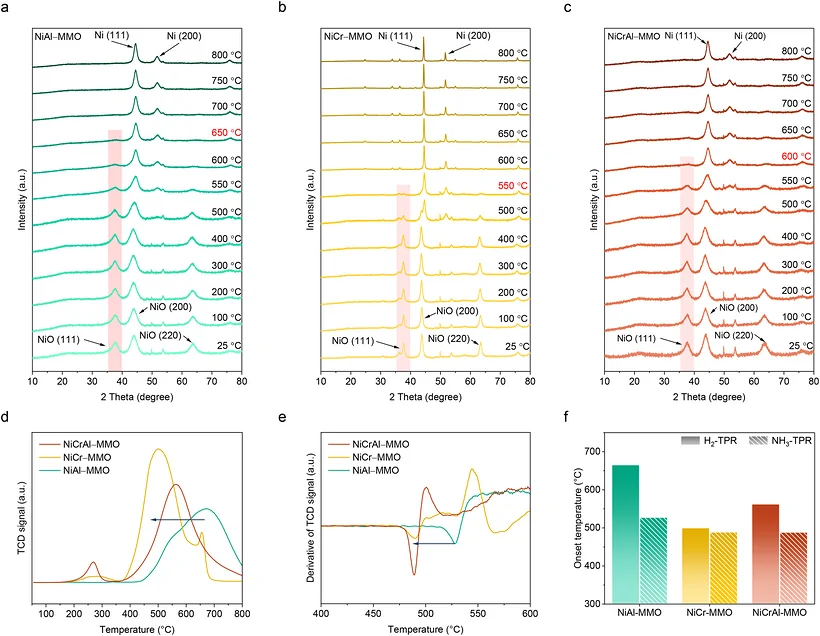
Charge‐transfer‐modulated reduction behavior of LDH‐derived MMO nanostructures. (a–c) In situ XRD patterns of (a) NiAl‐MMO, (b) NiCr‐MMO, and (c) NiCrAl‐MMO recorded under an H_2_ atmosphere. (d) H_2_‐TPR profiles of the MMO nanostructures. (e) Derivative NH_3_‐TPR profiles of the MMO nanostructures. (f) Comparison of the reduction‐onset temperatures derived from the H_2_‐TPR and NH_3_‐TPR profiles of the MMO nanostructures.

The same conclusion was reached from H_2_‐TPR analysis (Figure 4d), where the reduction peaks shifted progressively to lower temperatures in the order of NiAl‐MMO, NiCrAl‐MMO, and NiCr‐MMO. Thus, both in situ structural monitoring and temperature‐programmed reduction consistently show that Cr enhances the reducibility of Ni species. Because catalyst activation during ammonia decomposition occurs under pure NH_3_ rather than H_2_, we further carried out NH_3_‐TPR to evaluate the reduction behavior under a more realistic reaction atmosphere (Figure 4e and Figure S24). Because NH_3_ decomposition can overlap with the reduction response in the TCD signal, additional NH_3_‐TPR measurements of the pre‐reduced RMMO samples were performed as a control to distinguish the reduction‐related features (Figure S25). Again, the Cr‐containing samples exhibited lower reduction temperatures than the Cr‐free sample, confirming that the beneficial effect of Cr on reduction is maintained under NH_3_.

Interestingly, NiCrAl‐MMO showed the lowest reduction temperature under NH_3_. This result highlights that the combination of Cr‐mediated electronic promotion and Al‐enabled structural stabilization is particularly effective under the actual catalyst‐activation environment. As summarized in Figure 4f, Cr incorporation systematically lowers the reduction‐onset temperature under both H_2_ and NH_3_ atmospheres. These findings demonstrate that charge transfer from Cr to Ni has a direct functional consequence: it promotes the formation of reduced Ni species under reaction‐relevant conditions, thereby linking electronic‐state tuning to catalyst activation.

### 2.4 Evaluation of Ammonia Decomposition Performance

The catalytic activities of the synthesized catalysts toward NH_3_ decomposition were evaluated in a fixed‐bed reactor under a weight hourly space velocity (WHSV) of 20,000 mL g_cat_^−1^ h^−1^ over the temperature range of 350°C–650°C (Figure 5a and Figure S26). Among the catalysts examined, NiCr‐RMMO showed substantially lower activity, highlighting the essential role of Al as a support component for stabilizing the active phase. In contrast, among the Al‐containing catalysts, NiCrAl‐RMMO consistently outperformed NiAl‐RMMO, demonstrating the beneficial effect of Cr incorporation. Accordingly, to distinguish the influence of the electronic state of Ni from the structural characteristics of the catalysts, the turnover frequency (TOF) for NH_3_ decomposition was calculated (Figure S27). NiCrAl‐RMMO exhibited the highest TOF despite having a slightly lower BET surface area than NiAl‐RMMO, indicating that the Cr‐induced electronic modification of Ni enhances the intrinsic catalytic activity when sufficient surface accessibility is maintained. In contrast, NiCr‐RMMO showed the lowest Ni^0^ 2p_3/2_ binding energy but a relatively low TOF, indicating that electron enrichment alone is insufficient to determine the intrinsic activity of the exposed Ni sites. In light of the preceding electronic‐structure analyses, this enhancement can be attributed to Cr‐to‐Ni electron transfer, which generates more electron‐rich Ni active sites favorable for ammonia decomposition.

**FIGURE 5:**
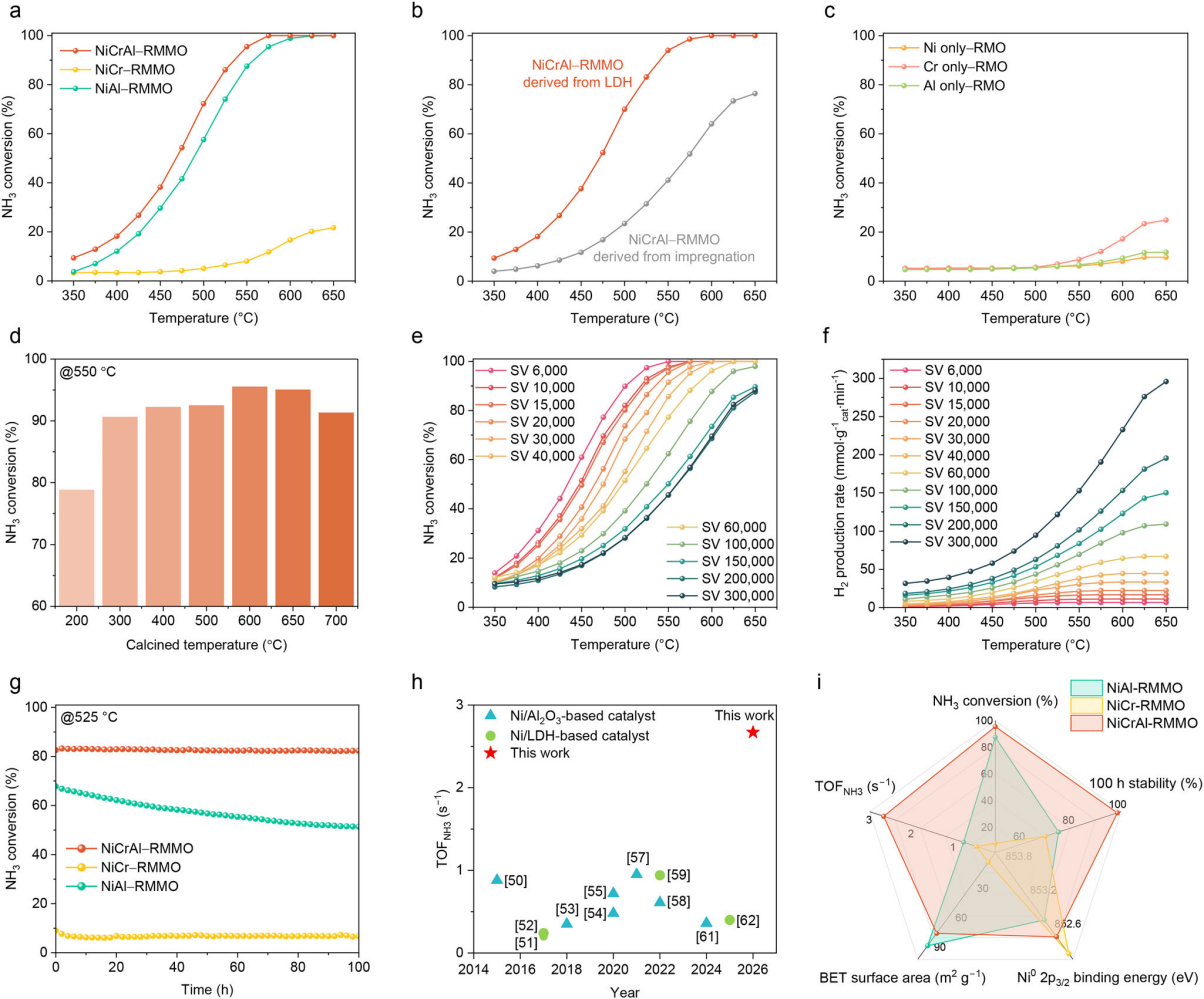
Catalytic performance of LDH‐derived mixed‐metal nanostructures for NH_3_ decomposition. (a) NH_3_ decomposition performance of NiAl‐RMMO, NiCr‐RMMO, and NiCrAl‐RMMO nanostructures (reaction conditions: 100% NH_3_, WHSV = 20,000 mL g_cat_^−1^ h^−1^). (b) Comparison of the catalytic performances of NiCrAl catalysts prepared by the LDH‐derived and impregnation methods. (c) NH_3_ decomposition performance of single‐component reduced metal oxide (RMO) control samples containing only Ni, Cr, or Al. (d) NH_3_ decomposition performance of NiCrAl‐RMMO nanostructures prepared using different calcination temperatures. (e) NH_3_ conversion as a function of WHSV. (f) H_2_ production rate as a function of WHSV. (g) Long‐term stability test of the RMMO catalysts over 100 h. (h) Comparison of TOF_NH3_ with previously reported Ni/Al_2_O_3_‐based catalysts for NH_3_ decomposition. Details of the literature comparison are provided in Table S3. (i) Radar‐chart comparison of the catalytic, structural, and electronic properties of the RMMO catalysts. The radar chart compares the NH_3_ conversion at 550°C, activity retention after 100 h, TOF_NH3_, BET specific surface area, and Ni^0^ 2p_3/2_ binding energy of NiAl‐RMMO, NiCr‐RMMO, and NiCrAl‐RMMO. For the Ni^0^ 2p_3/2_ binding‐energy axis, lower binding energies are plotted toward the outer direction, corresponding to increasing electron density of Ni.

To further assess the validity of the computational design strategy, we synthesized catalysts containing other elements identified by DFT screening and examined their catalytic activities after incorporation into the LDH framework. The experimental activity trend generally followed the DFT predictions, except for Co, which was predicted to withdraw electron density from Ni and did not improve the catalytic performance (Figure S28). We also optimized the elemental ratio within the NiCrAl system and found that the 6:1:1 composition exhibited the highest activity among the compositions investigated (Figure S29). These results further support the conclusion that rational tuning of the Ni electronic structure through promoter selection and composition control is an effective strategy for improving ammonia decomposition activity. Catalytic activities were further compared by varying the Cr/Al ratio within the NiCrAl system (Figure S30). Although the activity differences were modest, the 6:1:1 composition exhibited the highest activity, confirming it as the optimal composition among those examined.

To evaluate the intrinsic reaction kinetics with minimized mass‐transfer effects, catalytic tests were additionally conducted at a much higher WHSV of 300,000 mL g_cat_^−1^ h^−1^, and apparent activation energies were extracted from Arrhenius plots constructed from these data (Figure S31) [48]. NiCrAl‐RMMO exhibited a significantly lower activation energy than the other catalysts, indicating that the reaction proceeds more readily on the electronically optimized Ni sites. This result is consistent with the beneficial effect of Cr‐induced electronic modulation on the overall reaction energetics, while the contributions of individual elementary steps are discussed separately below based on DFT and kinetic analyses.

The structural advantage of the LDH‐derived catalyst platform was verified by comparing NiCrAl‐RMMO with a reference catalyst of identical overall composition prepared by conventional impregnation (Figure 5b). The impregnated catalyst showed markedly lower activity than the LDH‐derived counterpart, indicating a beneficial effect of the homogeneous cation distribution established through the LDH precursor route [49]. To clarify whether the performance enhancement originates from any single component alone, single‐element control samples containing only Ni, Cr, or Al were synthesized under the same conditions and tested for ammonia decomposition. All of these control samples exhibited negligible activity, confirming that the outstanding performance of NiCrAl‐RMMO arises from the cooperative interaction among Ni, Cr, and Al rather than from any individual component.

We next examined how synthetic parameters affect the catalytic performance of NiCrAl‐RMMO. The calcination temperature of the LDH precursor strongly influenced the activity (Figure 5d and Figure S32). The catalyst calcined at 200°C showed poor activity, whereas the activity increased progressively with calcination temperature and reached a maximum at 600°C. Upon further increasing the calcination temperature, the activity declined, which is attributed to excessive agglomeration of the active species. The progressive sharpening of the NiO diffraction peaks and the corresponding increase in the apparent NiO crystallite size with increasing calcination temperature further support thermally induced crystallite growth at elevated temperatures (Figure S33). The nature of the Ni precursor also had a significant effect: the catalyst prepared from Ni nitrate showed much higher activity than that prepared from Ni chloride, suggesting that residual Cl species may adversely affect the catalyst performance (Figure S34).

The catalytic behavior of NiCrAl‐RMMO was further investigated as a function of WHSV (Figure 5e,f). As the WHSV increased, the NH_3_ conversion gradually decreased because of the shortened residence time, whereas the H_2_ production rate increased owing to the higher overall NH_3_ throughput. Thus, although the fractional conversion decreases at higher feed rates, the total hydrogen productivity increases. The long‐term stability of the catalysts was assessed by a 100 h durability test at 525°C (Figure 5g and Figure S35). NiCrAl‐RMMO maintained stable catalytic activity throughout the 100 h test, whereas NiAl‐RMMO showed a clear decrease in absolute NH_3_ conversion. Although the decrease in NiCr‐RMMO appears less pronounced in the absolute conversion profile because of its intrinsically low initial activity, the normalized activity (*X/X_0_*) reveals a substantial relative loss of activity, which is even greater than that of NiAl‐RMMO (Figure S35). Post‐stability analyses indicate that the deactivation mechanisms differ between NiAl‐RMMO and NiCr‐RMMO. The activity loss of NiAl‐RMMO is associated in part with greater accumulation of surface nitrogen species, whereas the instability of NiCr‐RMMO is dominated by severe aggregation and CrN formation. Post‐stability XRD, TEM/EDS, XPS, and BET analyses further confirmed that NiAl‐RMMO and NiCrAl‐RMMO largely retained their phase structure, nanoscale dispersion, metallic Ni state, and surface area, whereas NiCr‐RMMO exhibited severe aggregation, CrN formation, and pronounced structural deterioration. Detailed post‐reaction characterization and discussion are provided in Figures S36–S39.

Finally, the performance of NiCrAl‐RMMO was benchmarked against previously reported Ni/Al_2_O_3_‐based catalysts for ammonia decomposition (Figure 5h and Table S3) [50, 51, 52, 53, 54, 55, 56, 57, 58, 59, 60, 61, 62, 63]. NiCrAl‐RMMO delivered an exceptionally high TOF_NH3_ of 2.67 s^−1^ and, within the comparison set considered here, showed the best performance among Ni/Al_2_O_3_‐based catalysts reported for NH_3_ decomposition. These results demonstrate that the superior catalytic behavior of NiCrAl‐RMMO originates from the synergistic combination of electronic promotion by Cr and structural stabilization by Al within the LDH‐derived mixed‐metal architecture. As further illustrated by the radar chart in Figure 5i, NiCr‐RMMO benefits from Cr‐induced electron enrichment but suffers from poor structural stabilization and low surface area in the absence of Al, whereas NiAl‐RMMO possesses a structurally favorable support environment but lacks the electronic promotion provided by Cr. In contrast, NiCrAl‐RMMO integrates both advantages, achieving an optimal balance between accessible Ni active sites and electronically enriched Ni species, which ultimately accounts for its superior catalytic performance.

### 2.5 Surface Reaction Kinetics of the RMMO Catalysts

To clarify the origin of the superior NH_3_ decomposition activity of NiCrAl‐RMMO, we investigated the surface reaction kinetics and the evolution of adsorbed intermediates on the RMMO catalysts. In situ diffuse reflectance infrared Fourier transform spectroscopy (DRIFTS) was first performed at room temperature to examine the initial NH_3_ adsorption behavior on each catalyst surface (Figure 6a–c). Lewis‐bound NH_3_ species (L‐NH_3_) were detected on all catalysts, whereas Brønsted‐bound NH_4_^+^ species (B‐NH_4_^+^) were additionally observed on NiAl‐RMMO [64]. Because NH_4_^+^ species on Brønsted acid sites require an additional dehydrogenation step before entering the main ammonia decomposition pathway, this adsorption mode is kinetically less favorable than NH_3_ adsorbed on Lewis acid sites [8, 65]. Although NiCr‐RMMO exhibited only Lewis acid sites, its overall NH_3_ adsorption was limited, which can be attributed to the absence of Al as a support component. By contrast, in NiCrAl‐RMMO, Cr incorporation converted the Brønsted acid sites present in NiAl‐RMMO into predominantly Lewis acid sites, resulting in a surface adsorption configuration that is more favorable for NH_3_ decomposition.

**FIGURE 6:**
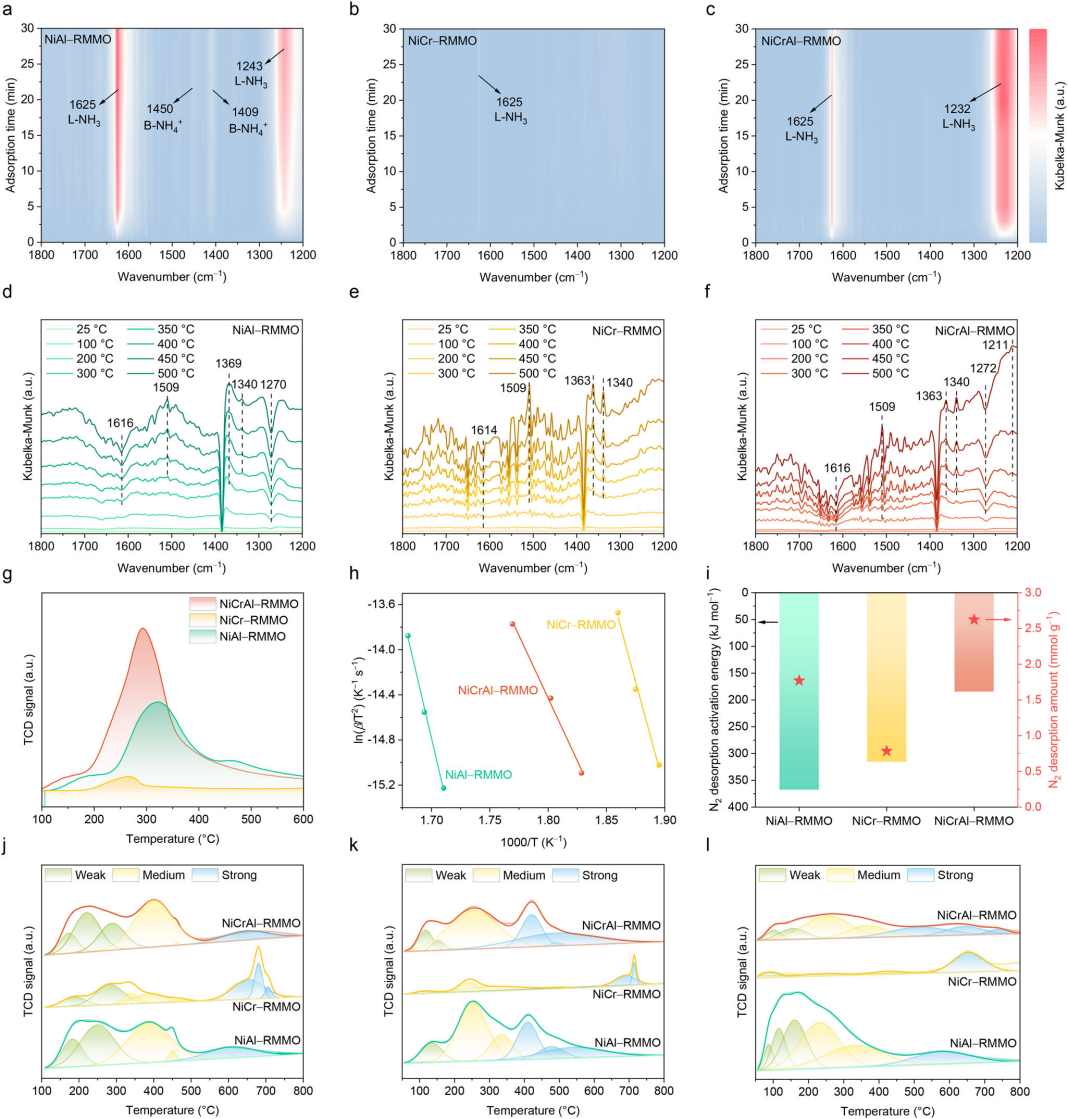
Mechanistic investigation of NH_3_ decomposition over LDH‐derived RMMO nanostructures. (a–c) In situ DRIFTS spectra recorded under 10% NH_3_/N_2_ over (a) NiAl‐RMMO, (b) NiCr‐RMMO, and (c) NiCrAl‐RMMO nanostructures at room temperature. (d–f) Temperature‐dependent in situ DRIFTS spectra recorded under 10% NH_3_/N_2_ over (d) NiAl‐RMMO, (e) NiCr‐RMMO, and (f) NiCrAl‐RMMO nanostructures. (g) N_2_ temperature‐programmed desorption (N_2_‐TPD) profiles of the RMMO nanostructures. (h) Redhead plots of ln(*β*/*T* ^2^) versus 1/*T*_m_ derived from N_2_‐TPD profiles of the RMMO nanostructures measured at different heating rates. (i) N_2_ desorption activation energies (*E*_des_) derived from the Redhead analysis of the N_2_‐TPD data, together with the total N_2_ desorption amounts of the RMMO nanostructures. (j–l) TPD profiles of the RMMO nanostructures: (j) NH_3_‐TPD, (k) CO_2_‐TPD, and (l) H_2_‐TPD.

To further monitor the evolution of surface intermediates during catalyst activation, temperature‐dependent in situ DRIFTS measurements were carried out during heating (Figure 6d–f and Figure S41). As the temperature increased, the intensities of the adsorbed NH_3_‐related bands at approximately 1616, 1614, and 1270 cm^−1^ gradually decreased, indicating that surface NH_3_ species were either thermally desorbed or consumed through progressive dehydrogenation [58, 66]. Concurrently, new bands corresponding to NH_2_* (amide) species at 1369, 1363, and 1340 cm^−1^ and NH* (imide) species at 1509 and 1508 cm^−1^ emerged with increasing temperature [67, 68, 69]. Notably, a bridged NH* species at 1211 cm^−1^ was distinctly observed only for NiCrAl‐RMMO at elevated temperatures. This bridged imide configuration allows the adsorbate to interact cooperatively with adjacent metal sites rather than being confined to a single site and may provide a geometry conducive to the final dehydrogenation step [70, 71, 72, 73].

Because nitrogen removal through N─N recombination and subsequent N_2_ desorption is widely recognized as a kinetic bottleneck in NH_3_ decomposition on non‐noble metal catalysts, we next carried out N_2_ temperature‐programmed desorption (N_2_‐TPD) analysis to quantitatively assess the N_2_ desorption component of this process (Figure 6g–i). The activation energy for N_2_ desorption (*E*_des_) was extracted using the Redhead model from N_2_‐TPD profiles collected at different heating rates (5–20°C min^−1^) (Figure 6g and Figure S42) [34, 74]. The calculated *E*_des_ decreased in the order of NiAl‐RMMO, NiCr‐RMMO, and NiCrAl‐RMMO, demonstrating that N_2_ desorption is most facile on NiCrAl‐RMMO. In particular, the substantially lower desorption activation energy of NiCrAl‐RMMO provides direct kinetic evidence for facilitated N_2_ desorption on this catalyst. It should be noted that N_2_‐TPD specifically probes N_2_ desorption and does not directly resolve the kinetics of N–N recombination; the latter therefore remains to be independently characterized. Accordingly, the present N_2_‐TPD results are interpreted specifically as evidence for facilitated N_2_ desorption. Moreover, NiCrAl‐RMMO exhibited the largest total N_2_ desorption amount, indicating the highest capacity for accommodating nitrogen‐containing surface species prior to desorption.

To further evaluate the possible roles of surface acid‐base properties and hydrogen adsorption behavior, we additionally performed NH_3_‐, CO_2_‐, and H_2_‐TPD analyses (Figure 6j–l and Figure S43 and Table S4). NH_3_‐TPD showed a modest increase in the number of acid sites for NiCrAl‐RMMO relative to NiAl‐RMMO, whereas CO_2_‐TPD indicated that the two catalysts possess similar amounts of basic sites. Interestingly, H_2_‐TPD revealed that NiCrAl‐RMMO has a markedly lower hydrogen adsorption capacity than NiAl‐RMMO. This contrast suggests that the superior NH_3_ decomposition performance of NiCrAl‐RMMO is unlikely to originate primarily from hydrogen adsorption/desorption behavior [6]. Instead, the catalytic advantage is more consistently explained by the optimized NH_3_ adsorption mode, the formation of kinetically favorable nitrogen‐containing intermediates, and the accelerated desorption of N_2_ from the catalyst surface.

### 2.6 Mechanistic Insights into the Enhanced Catalytic Activity: The Role of Electron‐Rich Ni Sites

Taken together, the superior NH_3_ decomposition performance of NiCrAl‐RMMO can be traced to the formation of electron‐rich Ni active sites induced by Cr incorporation. DFT calculations first identified Cr as the most effective promoter among the LDH‐compatible metal cations for donating electron density to Ni. Increasing the electron density around Ni was predicted to make NH_3_ activation more favorable while simultaneously weakening N_2_ binding, thereby facilitating product desorption. These results indicate that Cr serves not merely as a structural component, but as an electronic promoter that deliberately tunes the redox state and catalytic properties of Ni. This prediction is strongly supported by the experimental results: XANES, KPFM, zeta potential, ELNES, depth‐profiled XPS, and EPR analyses consistently showed that Cr incorporation increases the electron density of Ni (Figure 3). In particular, the progressive shift of the metallic Ni binding energy to lower values and the increase in Ni d‐orbital occupancy provide direct evidence for Cr‐to‐Ni charge transfer and the resulting formation of electron‐rich Ni sites.

The NH_3_ decomposition reaction proceeds through a sequence of elementary steps involving NH_3_ adsorption, stepwise dehydrogenation, formation of surface nitrogen intermediates, and finally N–N and H–H recombination followed by product desorption. The associated electron flow during this process is schematically summarized in Figure 7. The reaction begins with adsorption of NH_3_ through interaction between the lone pair of the N atom and vacant electronic states on the catalyst surface, forming adsorbed NH_3_ species on Lewis acid sites. In situ DRIFTS analysis showed that NH_3_ adsorption on NiCrAl‐RMMO occurs predominantly on Lewis acid sites, whereas NiAl‐RMMO also contains Brønsted acid sites that stabilize NH_4_^+^ species. Because NH_4_^+^ formation introduces an additional dehydrogenation requirement before entering the main decomposition pathway, the dominant Lewis‐acid adsorption mode on NiCrAl‐RMMO provides a kinetically more favorable starting point for NH_3_ decomposition (Figure 6a–c).

**FIGURE 7:**
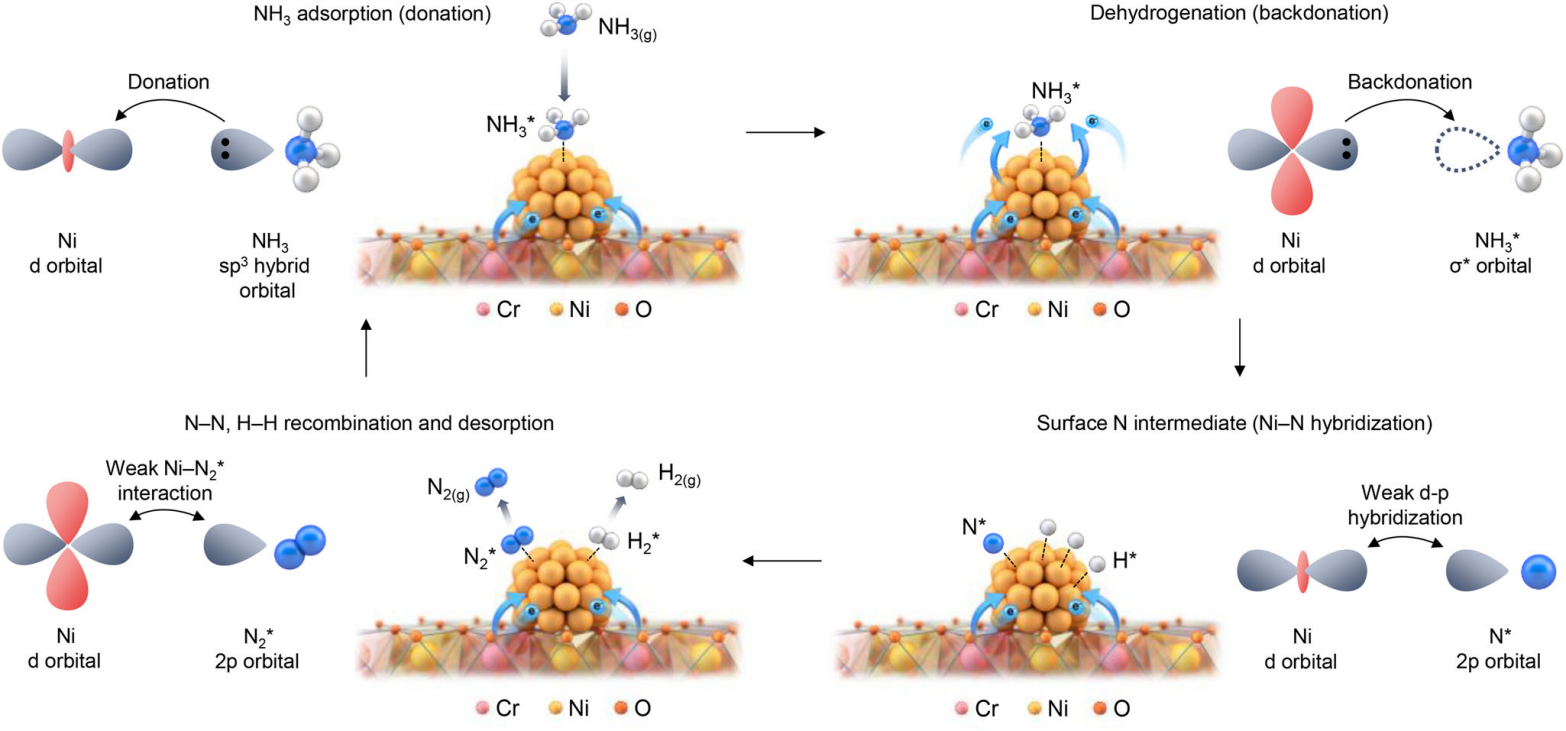
Proposed reaction mechanism for NH_3_ decomposition over electron‐rich Ni sites in the NiCrAl‐RMMO catalyst.

Following adsorption, the catalyst must promote sequential N─H bond cleavage to convert NH_3_* into NH_2_*, NH*, and ultimately N*. At this stage, the electron‐rich nature of Ni is expected to play a critical role by facilitating interaction with the antibonding orbitals of NH_3_‐derived intermediates, thereby favorably modifying the dehydrogenation energetics. This interpretation is further supported by the DFT‐calculated reaction‐energy profiles of the full NH_3_ decomposition pathway (NH_3_* → NH_2_* → NH* → N* → N_2_* → N_2_(g)), which show that increasing the electron density of Ni favorably modifies the energetics of NH_3_‐derived intermediates along the dehydrogenation pathway (Figure S3). These profiles represent DFT total‐energy differences between optimized intermediates and are therefore interpreted as qualitative reaction energetics rather than explicit activation barriers.

To provide additional kinetic context, we further examined the dependence of the NH_3_ consumption rate on NH_3_ partial pressure. The reaction order with respect to NH_3_ increased from 0.29 for NiAl‐RMMO to 0.41 for NiCrAl‐RMMO (Figure S44). The larger positive reaction order of NiCrAl‐RMMO indicates a stronger dependence of the reaction rate on NH_3_ partial pressure on the Cr‐promoted catalyst. Although the reaction‐order analysis alone does not identify a specific elementary rate‐determining step, its trend, together with the DFT reaction‐energy profiles and the NH_2_* and NH* intermediates observed by temperature‐dependent DRIFTS, is consistent with altered NH3‐derived surface chemistry and more favorable dehydrogenation energetics on electron‐rich Ni sites. Complementing these kinetic measurements, temperature‐dependent in situ DRIFTS measurements revealed the formation of NH_2_* and NH* species on NiCrAl‐RMMO, together with the distinct appearance of a bridged NH* intermediate at elevated temperatures (Figure 6d–f). This bridged imide configuration allows the adsorbate to interact cooperatively with adjacent metal sites rather than being confined to a single site and may provide a geometry conducive to the final dehydrogenation step. These observations are therefore consistent with a modified dehydrogenation pathway on the Cr‐modulated Ni surface.

After dehydrogenation, the resulting surface N* species must undergo N–N recombination and subsequent N_2_ desorption, which are widely recognized as important steps in nitrogen removal during NH_3_ decomposition over non‐noble metal catalysts. Electron enrichment weakens N_2_ binding, as indicated by the DFT adsorption energetics (Figure S45). In agreement with this picture, N_2_‐TPD analysis showed that NiCrAl‐RMMO possesses the lowest N_2_ desorption activation energy among the catalysts examined, indicating the most facile N_2_ desorption kinetics (Figure 6g–i). This result provides direct kinetic evidence that electronic tuning of Ni facilitates N_2_ release and thereby promotes nitrogen removal from the catalyst surface.

In summary, Cr incorporation creates electron‐rich Ni active sites that optimize the NH_3_ decomposition pathway at multiple stages. These sites favor NH_3_ adsorption in a reaction‐relevant Lewis‐acid configuration, facilitate sequential dehydrogenation from NH_3_* to NH_2_*, NH*, and N*, and promote nitrogen removal by weakening N_2_ binding and facilitating N_2_ desorption. The outstanding catalytic performance of NiCrAl‐RMMO therefore originates from electronic‐structure‐driven optimization of the entire reaction pathway rather than from a single isolated improvement. More broadly, these findings demonstrate that charge‐transfer engineering in LDH‐derived mixed‐metal nanostructures provides an effective strategy for designing high‐performance, Ru‐free catalysts for ammonia‐based hydrogen generation.

## 3 Conclusion

In conclusion, we have developed an LDH‐derived catalyst platform that couples structural stabilization of highly dispersed active species with deliberate electronic‐state engineering to unlock high‐performance ammonia decomposition over a non‐noble metal catalyst. Through the rational incorporation of Cr into a NiAl‐based framework, we demonstrate that Cr serves not simply as an additional component, but as an active electronic promoter that donates electron density to Ni and generates electron‐rich Ni active sites. This Cr‐to‐Ni charge transfer, predicted by DFT and consistently verified by multiple spectroscopic and microscopic analyses, provides the central mechanistic origin of the enhanced catalytic behavior. The electron‐rich Ni sites created in NiCrAl‐RMMO optimize the NH_3_ decomposition pathway at multiple levels: they favor NH_3_ adsorption in a reaction‐relevant Lewis‐acid mode, facilitate progressive N─H bond cleavage, enhance catalyst reducibility, and most critically, weaken N_2_ binding and facilitate N_2_ desorption. By combining Cr‐induced electronic promotion with Al‐enabled structural stabilization, NiCrAl‐RMMO achieves 95.5% NH_3_ conversion at 550°C and a high H_2_ production rate of 22.83 mmol g_cat_^−1^ min^−1^ at 500°C and a WHSV of 30,000 mL g_cat_^−1^ h^−1^, while maintaining stable catalytic performance over 100 h. Collectively, these results demonstrate that the exceptional activity and durability of NiCrAl‐RMMO arise from the synergistic combination of Cr‐to‐Ni charge transfer, enhanced Ni reducibility, and Al‐enabled stabilization of dispersed Ni active sites. In this regard, charge‐transfer engineering offers a powerful route to overcoming the intrinsic reactivity limitations of non‐noble metal catalysts. These findings provide a general blueprint for the development of next‐generation Ru‐free catalysts for ammonia‐to‐hydrogen conversion and other catalytic processes governed by metal‐centered electronic states.

## 4 Experimental Section

Experimental details are provided in the Supporting Information.

## Author Contributions

**Donghyeok Kim**: conceptualization, writing – original draft, investigation. **Su‐Jin Kim**: conceptualization, investigation. **Gayeong Kim**: conceptualization, writing – original draft, investigation. **Hyoseok Kim**: investigation. **Jongkyoung Kim**: investigation. **Myeung‐Jin Lee**: investigation. **Bora Jeong**: investigation. **Minkyu Kim**: investigation, writing – review and editing, supervision, funding acquisition. **Hong‐Dae Kim**: investigation, writing – review and editing, supervision, funding acquisition. **Seungho Cho**: investigation, writing – review and editing, supervision, funding acquisition.

## Conflicts of Interest

The authors declare no conflicts of interest.

## Supporting information




**Supporting File**: advs78184‐sup‐0001‐SuppMat.docx.

## Data Availability

The data that support the findings of this study are available from the corresponding author upon reasonable request.
