# Supplementary material for: Electron‐Rich Nickel Enabled by Charge Transfer in Multimetal Nanostructures for Efficient Ammonia‐to‐Hydrogen Conversion
Source: Adv Sci (Weinh). 2026 Oct 6:e78184. Online ahead of print. doi: 10.1002/advs.78184 (PMC13643117; doi:10.1002/advs.78184)
Supplement: Supplementary file 1 [file ADVS-9999-e78184-s001.docx]

Supporting information

Electron-Rich Nickel Enabled by Charge Transfer in Multimetal Nanostructures for Efficient Ammonia-to-Hydrogen Conversion

Donghyeok Kim, Su-Jin Kim, Gayeong Kim, Hyoseok Kim, Jongkyoung Kim, Myeung-Jin Lee, Bora Jeong, Minkyu Kim*, Hong-Dae Kim*, and Seungho Cho*

D. Kim, H. Kim, J. Kim, Prof. S. Cho
Department of Materials Science and Engineering
Ulsan National Institute of Science and Technology (UNIST)
Ulsan 44919, Republic of Korea
E-mail: scho@unist.ac.kr

D. Kim, S. Kim, M. Lee, B. Jeong, Dr. H. Kim
Ulsan Technology Application Division
Korea Institute of Industrial Technology (KITECH)
Ulsan 44413, Republic of Korea
E-mail: hdkim@kitech.re.kr

G. Kim, Prof. M. Kim
Department of Chemical Engineering
Yeungnam University
Gyeongsan 38541, Republic of Korea
E-mail: mk_kim@ynu.ac.kr

J. Kim, Prof. S. Cho
UNIST InnoCORE hydro*studio
Ulsan National Institute of Science and Technology (UNIST)
Ulsan 44919, Republic of Korea

Prof. S. Cho
Graduate School of Semiconductor Materials and Devices Engineering
Ulsan National Institute of Science and Technology (UNIST)
Ulsan 44919, Republic of Korea

**Experimental Section**

**Chemicals**

Ni(NO_3_)_2_·6H_2_O (99.999%) and NiCl_2_·6H_2_O (99.9%), Cr(NO_3_)_3_·9H_2_O (99%), Al(NO_3_)_3_·9H_2_O (99.99%), Co(NO_3_)_2_·6H_2_O (≥98%), Cu(NO_3_)_2_·3H_2_O (≥98%), Fe(NO_3_)_3_·9H_2_O (≥98%), Mn(NO_3_)_2_·4H_2_O (≥97%), and hexamethylenetetramine (HMTA, ≥99%) were purchased from Sigma-Aldrich. Deionized (DI) water was used as the solvent in all synthesis procedures. All chemicals were of analytical grade and used as received without further purification.

**Synthesis of Ni-Based LDHs**

Ni-based layered double hydroxides (LDHs) were synthesized by a one-pot hydrothermal method. Metal precursors were dissolved in DI water (0.125 M, 80 mL) under continuous stirring for 30 min. HMTA was then added to the solution with a precipitant-to-metal precursor molar ratio of 2.14, followed by additional stirring for 30 min. The resulting mixture was transferred to a 100 mL Teflon-lined autoclave and hydrothermally treated at 120 °C for 12 h. After the autoclave was allowed to cool naturally to room temperature, the precipitates were collected by centrifugation at 7000 rpm for 3 min, washed twice with DI water, and dried at 80 °C for 24 h.

**Preparation of NH_3_ Decomposition Catalysts**

The as-prepared LDH precursors were ground and calcined in air at 600 °C for 2 h to obtain the corresponding mixed-metal oxides (MMOs). The calcined MMOs were then reduced under flowing pure NH_3_ at 650 °C for 2 h to yield the reduced mixed-metal oxides (RMMOs).

**Materials Characterization**

The crystal structures and phase purities of the synthesized catalysts were investigated using a multi-purpose high-power X-ray diffractometer (MPXRD, SmartLab, Rigaku) with Cu Kα radiation (*λ* = 1.5406 Å). Diffraction data were collected over a 2*θ* range of 5–80° at a scanning rate of 2° min^−1^.

In situ XRD measurements were performed using the same diffractometer equipped with a high-temperature reaction chamber to monitor phase transformations under different atmospheres. To investigate the structural evolution from the LDH precursor to the MMO phase, in situ XRD patterns were recorded under air while heating the samples from 25 to 800 °C at a ramping rate of 25 °C min^−1^. Diffraction data were collected at 25, 100, 200, 300, 400, 500, 550, 600, 650, 700, 750, and 800 °C. At each temperature, two consecutive scans were acquired to ensure structural stability. Each scan was recorded over 2*θ* = 5–80° with a scan speed of 2° min^−1^ and a step width of 0.02°. To examine the reduction behavior from MMO to RMMO, in situ XRD was also conducted under a reducing atmosphere of 10% H_2_/Ar (50 mL min^−1^). The temperature program and measurement sequence were identical to those used under air.

The surface morphologies and microstructures of the synthesized catalysts were examined using field-emission scanning electron microscopy (FE-SEM, SU8020, Hitachi) at an accelerating voltage of 10 kV.

Spherical aberration-corrected scanning transmission electron microscopy (Cs-corrected STEM) was performed on a JEM-ARM300F microscope (JEOL) operated at 300 kV to examine the morphology and dispersion of active species at high magnification. Elemental distributions were analyzed by energy-dispersive X-ray spectroscopy (EDS) mapping. In addition, electron energy-loss spectroscopy (EELS) was used to obtain electron-loss near-edge structure (ELNES) spectra. The Ni L_2,3_-edge ELNES spectra were specifically analyzed to probe the electronic state and d-band occupancy of Ni species in the catalysts.

The elemental compositions and metal loadings of the synthesized nanostructures were determined by inductively coupled plasma optical emission spectroscopy (ICP-OES, 700-ES, Varian).

The specific surface areas and pore size distributions were determined from N_2_ adsorption–desorption isotherms measured at −196 °C using a TriStar 3020 analyzer (Micromeritics). Before measurement, all samples were degassed at 150 °C for 1 h under vacuum to remove adsorbed moisture and impurities. The specific surface areas were calculated by the Brunauer–Emmett–Teller (BET) method, and the pore size distributions were derived from the adsorption branches of the isotherms using the Barrett–Joyner–Halenda (BJH) model.

X-ray absorption near-edge structure (XANES) and extended X-ray absorption fine structure (EXAFS) analyses were carried out at the Ni K-edge (8333 eV) on the BL 6D beamline at the Pohang Accelerator Laboratory (PAL). XAFS spectra were acquired in transmission mode, and Ni foil and NiO were used as standard references for energy calibration.

The surface potential distributions of the nanostructures were investigated by atomic force microscopy (AFM, XE-700, Park Systems). Kelvin probe force microscopy (KPFM) measurements were performed in non-contact mode using a conductive tip. The surface charge characteristics of the nanostructures were further evaluated by zeta-potential measurements (ZetaPALS, Brookhaven Instruments) in DI water. The zeta potentials were measured at pH 7. Each measurement was repeated 20 times, and the average value was reported.

X-ray photoelectron spectroscopy (XPS) measurements were carried out on an AXIS Supra instrument (KRATOS Analytical Ltd.) using a monochromated Al K*α* X-ray source (*hν* = 1486.6 eV). To probe the chemical state of metallic Ni beneath the surface oxide layer, depth profiling was performed using Ar^+^ ion sputtering for 0, 30, 60, and 120 s. Because Ar^+^ sputtering may induce partial reduction of Ni species, the depth-profiled spectra were compared at matched etching times. The relative Ni 2p_3/2_ binding-energy difference between NiAl-RMMO and NiCrAl-RMMO was maintained across 30, 60, and 120 s of etching (Figure S17). Accordingly, the depth-profiled XPS results are interpreted primarily in terms of relative intersample binding-energy differences at matched sputtering times, while a minor contribution from sputter-induced reduction cannot be completely excluded. The sputtering rate was calibrated to 8 nm min^−1^ using a Ta_2_O_5_ reference standard. All binding energies were calibrated against the C 1s peak at 284.8 eV to compensate for surface charging.

The presence and behavior of unpaired electrons were examined using an electron paramagnetic resonance spectrometer (EPR, EMXplus, Bruker). EPR spectra were recorded at room temperature.

Temperature-programmed desorption (TPD) and temperature-programmed reduction (TPR) measurements were performed using a Microtrac BELCAT II instrument. All TPD (NH_3_-, CO_2_-, and H_2_-TPD) and TPR (H_2_-TPR and NH_3_-TPR) measurements were conducted under identical experimental conditions for consistency. Before each measurement, the samples were pretreated at 150 °C for 1 h under N_2_ to remove adsorbed moisture and surface impurities. The TPD and TPR profiles were then recorded while heating the samples to the target temperature at a constant ramping rate of 20 °C min^−1^.

CO pulse chemisorption measurements were performed to determine the Ni dispersion of the RMMO catalysts. Prior to measurement, the samples were reduced under 10% H_2_ at 500 °C for 30 min, followed by purging with N_2_. CO was then introduced in successive pulses, and the amount of chemisorbed CO was used to determine the Ni dispersion.

**In situ DRIFTS measurements**

In situ diffuse reflectance infrared Fourier transform spectroscopy (DRIFTS) experiments for NH_3_ decomposition and CO adsorption were carried out using a Bruker Vertex 70v spectrometer equipped with an in situ high-vacuum DRIFTS reaction cell (Harrick Scientific Products) and a liquid-nitrogen-cooled mercury cadmium telluride (MCT) detector. Spectra were collected by averaging 32 scans at a resolution of 4 cm^−1^ and are presented in Kubelka–Munk units.

For NH_3_ decomposition DRIFTS experiments, freshly prepared RMMO samples were loaded into the in situ chamber and treated under 10% NH_3_/N_2_ at 500 °C for 2 h. The samples were then purged with N_2_ at 500 °C for 1 h to remove surface ammonia, and the background spectrum was recorded at room temperature. After background collection, 10% NH_3_/N_2_ (50 mL min^−1^) was introduced into the reaction chamber for 30 min, and the spectra were recorded. For temperature-dependent in situ DRIFTS analysis, spectra were collected at 25, 100, 200, 300, 350, 400, 450, and 500 °C. At each temperature, the sample was held for 20 min to ensure steady-state conditions before data acquisition.

For CO adsorption measurements, the reduction pretreatment and background collection were performed under the same conditions as those used for NH_3_-DRIFTS. Subsequently, 1% CO/N_2_ (50 mL min^−1^) was introduced into the chamber for 30 min, and the spectra were then recorded.

**Catalytic Activity Evaluation**

Catalytic activity tests were conducted in a quartz fixed-bed reactor (inner diameter: 12.7 mm; length: 400 mm) under atmospheric pressure. For the standard catalytic activity tests, 60 mg of catalyst was packed into the reactor. Before catalytic evaluation, the catalyst was pretreated under pure NH_3_ by heating to 650 °C and holding at that temperature for 2 h for reduction and activation. The catalytic activity was then evaluated over a temperature range of 350–650 °C with a temperature interval of 25 °C. The weight hourly space velocity (WHSV) was varied from 6,000 to 300,000 mL g_cat_^−1^ h^−1^ to examine performance under different flow conditions. For the WHSV-dependent measurements shown in Figure 5e,f, both the catalyst loading and inlet NH_3_ flow rate were adjusted according to the target WHSV. Specifically, catalyst loadings of 0.03–0.20 g and pure NH_3_ flow rates of 20–200 mL min^−1^ were used to achieve WHSV values ranging from 6,000 to 300,000 mL g_cat_^−1^ h^−1^. The outlet gas composition, specifically the NH_3_ concentration, was analyzed online by using a gas chromatography (GC) equipped with a thermal conductivity detector (TCD). Considering the increase in the total gas volume caused by NH_3_ decomposition (2NH_3_ → N_2_ + 3H_2_), the NH_3_ conversion (X_NH3_) was calculated using the volume-corrected equation. The NH_3_ conversion ($X_{\mathrm{NH}_{3}}$) and H_2_ formation rate ($\gamma_{H_{2}}$) were calculated according to Equations (S1) and (S2):

$X_{\mathrm{NH}_{3}}= \frac{(1- C_{{NH}_{3,out}})}{{(1+ C}_{\mathrm{NH}_{3},out})}$	(S1)

$\gamma_{H_{2}} \left( \mathrm{mmol}{g_{\mathrm{cat}}}^{-1} \min^{-1} \right)= \frac{V_{\mathrm{NH}_{3}}\times X_{\mathrm{NH}_{3}}\times1.5}{22.4 \times M_{\mathrm{cat}}}$	(S2)

where $C_{{NH}_{3},in}$ and $C_{{NH}_{3},out}$ are the inlet and outlet NH_3_ concentrations, respectively, $V_{{NH}_{3}}$ is the NH_3_ flow rate (mL min^−1^), and *M*_cat_ is the catalyst mass (g). The H_2_ production rates presented in Figure 5f were calculated using Equation (S2) based on the inlet NH_3_ flow rate, measured NH_3_ conversion, and catalyst mass.

For the stability test, after pretreatment at 650 °C for 2 h under pure NH_3_, the outlet NH_3_ concentration was monitored for 100 h on stream at 525 °C and a WHSV of 20,000 mL g_cat_^−1^ h^−1^.

To further examine the intrinsic catalytic activity, the apparent activation energy (*E*_a_) was determined from Arrhenius plots. Kinetic measurements were performed under differential reactor conditions, where the NH_3_ conversion was maintained below 15% to minimize the influence of heat and mass transfer limitations and ensure a reaction-controlled regime.

The NH_3_ consumption rate (r_NH3_) used for the Arrhenius analysis was calculated from the NH_3_ conversion according to Equation (S3):

$r_{{NH}_{3}} \left( mmol {g_{cat}}^{-1} {min}^{-1} \right)=\frac{(V_{{NH}_{3}}\times X_{{NH}_{3}})}{(22.4 \times M_{cat})}$	(S3)

where V_NH3_ is the inlet NH_3_ flow rate (mL min^−1^), X_NH3_ is the NH_3_ conversion, and M_cat_ is the catalyst mass (g). At each reaction temperature, the outlet NH_3_ concentration was monitored until a steady state was reached, and the corresponding steady-state NH_3_ conversion was used to calculate r_NH3_. The apparent activation energy was obtained from the slope of the linear fit of ln(r_NH3_) versus 1/T according to the Arrhenius relationship:

$\ln\left( r_{{NH}_{3}} \right)=\ln\left( A \right)-\frac{E_{a}}{RT}$	(S4)

where A is the apparent pre-exponential factor, R is the ideal gas constant, and T is the absolute temperature (K).

NH_3_ reaction-order measurements were performed at 500 °C using 30 mg of catalyst under a total flow rate of 100 mL min^−1^. The NH_3_ partial pressure was varied by adjusting the NH_3_/N_2_ flow rates to 8/92, 12/88, 20/80, and 30/70 mL min^−1^, while maintaining a constant total flow rate. The NH_3_ consumption rate (r_NH3_) was calculated from the steady-state NH_3_ conversion. The apparent reaction order with respect to NH_3_ was determined from the slope of the linear relationship between ln(r_NH3_) and ln(P_NH3_).

**Computational Details**

All density functional theory (DFT) calculations were performed using the Vienna Ab initio Simulation Package (VASP) with the projector-augmented wave (PAW) method.^[S1-S3]^ Exchange–correlation interactions were described using the Perdew–Burke–Ernzerhof (PBE) functional within the generalized gradient approximation (GGA). All calculations were performed without spin polarization (ISPIN = 1). No empirical dispersion correction (e.g., D2, D3, or D4) was applied in the present calculations. Accordingly, the computational results are discussed primarily in terms of relative trends among model systems treated consistently within the same computational framework. Bulk structures were fully optimized prior to slab construction. Ni was modeled in its face-centered cubic (fcc) phase (space group Fm–3m) with an optimized lattice constant of *a* = *b* = *c* = 3.517 Å.^[S4]^ *γ*-Al_2_O_3_ was modeled using a monoclinic bulk structure (space group P2_1_/m) with lattice parameters *a* = *b* = 4.805 Å and *c* = 13.115 Å.^[S5]^ Brillouin-zone sampling for bulk calculations employed an 8 × 8 × 8 Monkhorst–Pack grid with a plane-wave kinetic-energy cutoff of 600 eV.

Surface models were constructed from the optimized bulk lattices. The Ni(111) slab for electron-count–tuning calculations was built using a 4 × 4 surface supercell with a slab thickness of 6.08 Å. The *γ*-Al_2_O_3_ surface slab was constructed as a 4 × 4 surface supercell with a slab thickness of 9.66 Å. A vacuum spacing of approximately 20 Å was applied along the surface-normal direction to avoid interactions between periodic images. Atomic positions were relaxed until the residual forces were below 0.05 eV Å^−1^. Each slab consisted of four atomic layers, and the bottom two layers were fixed at their bulk positions. For slab and cluster simulations, a plane-wave cutoff of 400 eV was employed, and Brillouin-zone sampling used a 2 × 2 × 1 Monkhorst–Pack grid.

For selected reaction-energy calculations on the Ni(111) surface, the total number of electrons was systematically varied using the NELECT parameter in the VASP INCAR file to impose controlled electron enrichment or depletion relative to the neutral slab. A compensating homogeneous background charge was introduced for the resulting charged periodic cell. For the reaction-energy profiles, the ΔE values were obtained from DFT total-energy differences between optimized intermediates. Explicit transition states and zero-point/thermal free-energy corrections were not evaluated; accordingly, these profiles are used to compare qualitative reaction energetics rather than quantitative activation barriers.

To model catalyst-relevant interfacial environments, supported model systems were constructed by placing a Ni_10_ cluster on the *γ*-Al_2_O_3_ slab and introducing Cr-containing interfacial modifiers at the Ni contact region. The geometry of the Ni_10_ cluster was not arbitrarily chosen; rather, it was taken from a published database of low-energy, atomically precise nanoclusters, in which diverse candidate cluster isomers were generated, fully relaxed by DFT, and ranked by total energy to identify the most stable structures.^[S6]^ From this energy-ranked set, the lowest-energy Ni_10_ isomer was selected and consistently used for all supported and unsupported model systems.

**Figure S1.** Support-free Ni–Cr cluster models used to rule out support-induced polarization effects and the corresponding Mulliken charges of the Ni cluster: (a) Cr0, (b) Cr1, (c) Cr2, and (d) Cr3.

**Figure S2.** Charge transfer in spinel-supported Ni_10_ cluster models. (a) Optimized structures and Mulliken charges of the Ni_10_ cluster supported on NiAl_2_O_4_ with zero to three interfacial Cr atoms. (b) Optimized structures and Mulliken charges of the Ni_10_ cluster supported on NiCr_2_O_4_ with zero, two, and four interfacial Cr atoms. (c, d) pDOS of the interfacial Ni site for the Ni-only and Ni–3Cr clusters on NiAl_2_O_4_; the Ni d-band center shifts from −0.790 to −0.804 eV upon Cr incorporation.

To verify that the Cr-to-Ni charge transfer identified on the γ-Al_2_O_3_-supported models remains operative in the spinel phases present in the experimental catalysts, additional model systems were constructed in which the Ni_10_ cluster was supported directly on the NiAl_2_O_4_ and NiCr_2_O_4_ spinel surfaces, and Cr atoms were progressively introduced at the metal–support interface (Figure S2). The spinel slab models were constructed from their fully optimized bulk structures, following the same computational procedure described above.

The Mulliken charge analysis consistently reproduces the charge-transfer behavior identified in the main text. On the NiAl_2_O_4_ surface, the Mulliken charge of the supported Ni_10_ cluster progressively decreases from +3.11 e for the Cr-free interface to +3.01, +2.77, and +2.63 e upon introducing one, two, and three interfacial Cr atoms, respectively (Figure S2a), demonstrating that the Ni cluster becomes increasingly electron-rich as the interfacial Cr content increases. The same monotonic behavior is observed on the NiCr_2_O_4_ spinel surface, where the Mulliken charge of the Ni cluster decreases from +2.02 e for the Cr-free interface to +1.99 and +1.93 e upon introducing two and four interfacial Cr atoms, respectively (Figure S2b).

Notably, the Ni_10_ cluster also carries a lower Mulliken charge on the NiCr_2_O_4_ support (+2.02 e) than on the NiAl_2_O_4_ support (+3.11 e) in the absence of additional interfacial Cr atoms. Because these models contain different spinel supports, this cross-support comparison is interpreted qualitatively and is consistent with stronger electron donation to Ni in the Cr-containing spinel environment. This observation is particularly relevant because NiCr_2_O_4_ is precisely the spinel phase identified in the NiCrAl-RMMO catalyst.

The electronic-structure analysis further supports this picture: on NiAl_2_O_4_, the pDOS of the interfacial Ni site shows a downward shift of the d-band center from −0.790 eV (Ni-only cluster) to −0.804 eV (Ni–3Cr cluster) (Figure S2c,d), consistent with the increased d-band filling identified for the γ-Al_2_O_3_-supported models in the main text (Figure 1b,c).

Taken together, these results confirm that the Cr-to-Ni charge transfer identified on the γ-Al_2_O_3_-supported models remains fully operative in the spinel-like environments (NiAl_2_O_4_ and NiCr_2_O_4_) present in the experimental catalysts.

**Figure S3.** Electronic-state dependence of NH_3_ decomposition on Ni and NiCr_2_O_4_. The profiles show DFT total-energy differences (ΔE) between optimized intermediates; explicit transition states and zero-point/thermal free-energy corrections were not included. Thus, the profiles are interpreted as qualitative reaction energetics rather than direct activation barriers.

**Table S1**. Elemental compositions of Ni, Cr, and Al in the synthesized LDH, MMO, and RMMO nanostructures, quantified by ICP-OES.

|  | Ni | | Cr | | | Al | | |
| --- | --- | --- | --- | --- | --- | --- | --- | --- |
|  | at.% | wt.% | | at.% | wt.% | | at.% | wt.% |
| NiAl-LDH | 67.64 | 81.97 | | – | – | | 32.36 | 18.03 |
| NiAl-MMO | 67.04 | 81.57 | | – | – | | 32.96 | 18.43 |
| NiAl-RMMO | 67.04 | 81.57 | | – | – | | 32.96 | 18.43 |
| NiCr-LDH | 75.56 | 77.73 | | 24.44 | 22.27 | | – | – |
| NiCr-MMO | 73.99 | 76.25 | | 26.01 | 23.75 | | – | – |
| NiCr-RMMO | 74.32 | 76.56 | | 25.68 | 23.44 | | – | – |
| NiCrAl-LDH | 67.33 | 75.25 | | 16.74 | 16.57 | | 15.93 | 8.18 |
| NiCrAl-MMO | 67.78 | 75.56 | | 16.69 | 16.48 | | 15.53 | 7.96 |
| NiCrAl-RMMO | 66.65 | 74.56 | | 17.40 | 17.24 | | 15.95 | 8.20 |

**Figure S4.** XRD patterns of NiAl-RMMO and NiCrAl-RMMO nanostructures.

**Figure S5.** TEM images of NiAl-RMMO, NiCr-RMMO, and NiCrAl-RMMO nanostructures.

**Table S2**. Textural properties, Ni dispersion, Ni crystallite size, and XRD-derived crystallinity of the synthesized MMO and RMMO nanostructures, determined by N_2_ sorption analysis, CO pulse chemisorption, and XRD analysis, respectively.

|  | S_BET_ (m^2^ g^−1^) | V_P_ (cm^3^ g^−1^) | D_P_ (nm) | Ni dispersion (%) | Crystallite size (nm) | Crystallinity (%) |
| --- | --- | --- | --- | --- | --- | --- |
| NiAl-MMO | 132 | 0.32 | 10.2 | - | - | - |
| NiAl-RMMO | 96.1 | 0.25 | 11.6 | 1.34 | 13.0 | 90.0 |
| NiCr-MMO | 32.1 | 0.09 | 12.3 | - | - | - |
| NiCr-RMMO | 11.0 | 0.01 | 51.7 | 0.18 | 42.7 | 100 |
| NiCrAl-MMO | 142 | 0.23 | 5.80 | - | - | - |
| NiCrAl-RMMO | 83.5 | 0.22 | 10.2 | 0.75 | 11.7 | 81.1 |

**Figure S6.** TEM images of (a) NiAl-LDH, (b) NiCr-LDH, and (c) NiCrAl-LDH. HAADF-STEM images and corresponding EDS elemental maps of the (d) NiAl-LDH, (e) NiCr-LDH, and (f) NiCrAl-LDH.

**Figure S7.** TEM images of (a) NiAl-MMO, (b) NiCr-MMO, and (c) NiCrAl-MMO. HAADF-STEM images and corresponding EDS elemental maps of the (d) NiAl-MMO, (e) NiCr-MMO, and (f) NiCrAl-MMO.

**Figure S8.** SEM images of LDH samples.

**Figure S9.** SEM images of MMO samples.

**Figure S10.** SEM images of RMMO samples.

**Figure S11.** *k*^2^-weighted Fourier-transform EXAFS spectra of the MMO nanostructures.

**Figure S12.** (a–c) Experimental Fourier-transform EXAFS spectra and corresponding fitting curves in R space for (a) NiAl-RMMO, (b) NiCr-RMMO, and (c) NiCrAl-RMMO. (d–f) Corresponding WT-EXAFS contour maps of (d) NiAl-RMMO, (e) NiCr-RMMO, and (f) NiCrAl-RMMO, showing the dominant Ni–Ni scattering contribution.

**Figure S13.** Surface potential profiles measured along the selected lines on the RMMO nanostructures and highly oriented pyrolytic graphite (HOPG) used as a reference sample.

**Figure S14.** HAADF-STEM images showing the positions selected for EELS line-scan analysis and the corresponding Ni L_2,3_-edge ELNES spectra of (a,b) NiAl-RMMO, (c,d) NiCr-RMMO, and (e,f) NiCrAl-RMMO. The hatched regions indicate the post-edge background ranges used for normalization of the white-line intensities.

Quantitative analysis of the Ni L_2,3_-edge white-line intensity is sensitive to the local specimen thickness. For NiAl-RMMO and NiCrAl-RMMO, sufficiently thin and relatively uniform regions could be selected for EELS measurements, allowing a meaningful comparison under comparable thickness conditions. In contrast, NiCr-RMMO exhibited severe aggregation and substantially thicker regions in the TEM specimen, making it difficult to obtain ELNES spectra under thickness conditions directly comparable to those of the Al-containing samples. Therefore, although the NiCr-RMMO ELNES spectrum is provided in Figure S14 for completeness, it was excluded from the quantitative d-band occupancy comparison in the main text to avoid potential bias arising from specimen-thickness effects.

**Figure S15.** Schematic illustration of the XPS depth-profiling procedure. Because the surface of Ni-based metal nanostructures is easily oxidized upon air exposure, Ar-ion etching was performed to remove the native oxide layer prior to XPS analysis, thereby enabling characterization of the underlying metallic region.

**Figure S16.** XPS depth profiles showing the elemental concentration changes as a function of etching time for (a) NiAl-RMMO, (b) NiCr-RMMO, and (c) NiCrAl-RMMO. The relative atomic concentrations of O 1s, Ni 2p, N 1s, Cr 2p, and C 1s were monitored during Ar-ion etching.

**Figure S17.** Depth-profiled Ni 2p XPS spectra of (a) NiAl-RMMO, (b) NiCr-RMMO, and (c) NiCrAl-RMMO recorded as a function of Ar-ion etching time (surface, 30, 60, and 120 s).

**Figure S18.** Depth-profiled Cr 2p XPS spectra of (a) NiAl-RMMO, (b) NiCr-RMMO, and (c) NiCrAl-RMMO recorded as a function of Ar-ion etching time (surface, 30, 60, and 120 s).

**Figure S19.** Depth-profiled Al 2p XPS spectra of (a) NiAl-RMMO, (b) NiCr-RMMO, and (c) NiCrAl-RMMO recorded as a function of Ar-ion etching time (surface, 30, 60, and 120 s).

**Figure S20.** Depth-profiled N 1s XPS spectra of (a) NiAl-RMMO, (b) NiCr-RMMO, and (c) NiCrAl-RMMO recorded as a function of Ar-ion etching time (surface, 30, 60, and 120 s).

**Figure S21.** Depth-profiled Cr 2p and Al 2p XPS spectra of the RMMO nanostructures.

**Figure S22.** Ni 2p_3/2_ XPS spectra of NiCrAl-RMMO catalysts with different Ni:Cr:Al ratios.

**Figure S23.** In situ XRD patterns of (a) NiAl-LDH, (b) NiCr-LDH, and (c) NiCrAl-LDH under an air atmosphere.

**Figure S24.** NH_3_-TPR profiles of the MMO nanostructures.

**Figure S25.** NH_3_-TPR analysis of the RMMO nanostructures: (a) TCD signal profiles and (b) corresponding first-derivative profiles of NiAl-RMMO, NiCr-RMMO, and NiCrAl-RMMO. The derivative profiles highlight the characteristic temperature regions associated with the rapid onset of NH_3_ decomposition over the pre-reduced RMMO catalysts.

**Figure S26.** Photograph of the fixed-bed reactor system used for catalytic performance evaluation.

**Figure S27.** Correlation of catalytic activity with the structural and electronic properties of the RMMO catalysts. (a) turnover frequencies for NH_3_ decomposition (TOF_NH3_) at 500 °C, calculated based on the amount of surface-exposed Ni determined from the measured Ni dispersion. (b) BET specific surface areas and Ni^0^ 2p_3/2_ binding energies of NiAl-RMMO, NiCr-RMMO, and NiCrAl-RMMO. The Ni^0^ 2p_3/2_ binding energies were determined from the depth-profiled XPS spectra after 30 s of Ar^+^ etching.

**Figure S28.** Catalytic performance of NiAl-based catalysts incorporating elements identified by DFT calculations as potential promoters in the LDH framework.

**Figure S29.** Catalytic performance of NiCrAl-RMMO catalysts with different Ni:Cr:Al ratios for NH_3_ decomposition: (a) NH_3_ conversion profiles and (b) magnified view of the 450–550 °C range to highlight the differences in activity.

**Figure S30.** Catalytic performance of NiCrAl-RMMO catalysts with different Ni:Cr:Al ratios for NH_3_ decomposition. The Ni content was fixed while the relative Cr/Al ratio was systematically varied (Ni:Cr:Al = 6:1.5:0.5, 6:1:1, and 6:0.5:1.5). The catalytic activities were evaluated under a relatively high WHSV of 40,000 mL g_cat_^−1^ h^−1^ to more clearly distinguish the composition-dependent activity differences. Reaction conditions: 100% NH_3_, WHSV = 40,000 mL g_cat_^−1^ h^−1^.

**Figure S31.** Evaluation of intrinsic reaction kinetics for NH_3_ decomposition over RMMO catalysts: (a) NH_3_ conversion profiles measured at a high space velocity (WHSV = 300,000 mL g_cat_^−1^ h^−1^) to minimize mass-transfer effects, (b) Arrhenius plots derived from the catalytic data, and (c) the calculated activation energies.

**Figure S32.** NH_3_ conversion profiles of NiCrAl-RMMO catalysts calcined at different temperatures.

**Figure S33.** (a) In situ XRD patterns of NiCrAl-LDH recorded under an air atmosphere while holding the sample for 2 h at each temperature. (b) Apparent NiO crystallite sizes estimated from the NiO (200) reflection using the Scherrer equation as a function of calcination temperature.

**Figure S34.** Catalytic performance of NiCrAl-RMMO catalysts prepared using different Ni precursors (nitrate and chloride) for NH_3_ decomposition.

**Figure S35.** Relative activity (*X*/*X*_0_) plots from the long-term stability test of RMMO catalysts for NH_3_ decomposition.

**Figure S36.** XRD patterns of (a) NiAl-RMMO, (b) NiCr-RMMO, and (c) NiCrAl-RMMO before and after the 100 h stability test.

The structural stability of the RMMO catalysts after the 100 h durability test was further examined by XRD (Figure S36). For NiAl-RMMO and NiCrAl-RMMO, no significant phase transformation was observed after the long-term reaction. The diffraction peaks corresponding to metallic Ni were largely preserved, while only minor changes in the support-related phases were detected. Specifically, the contribution from amorphous alumina slightly decreased, accompanied by a somewhat more pronounced spinel-related diffraction feature after the stability test. These results indicate that the overall phase structures of the Al-containing catalysts were well maintained during prolonged NH_3_ decomposition.

In contrast, NiCr-RMMO exhibited a pronounced structural reconstruction after 100 h of reaction. Whereas the fresh NiCr-RMMO consisted primarily of metallic Ni and Cr_2_O_3_ phases, the spent catalyst showed diffraction features attributable to metallic Ni and CrN, indicating a substantial transformation of the Cr-containing phase during prolonged exposure to NH_3_. This pronounced phase evolution is likely associated with the poor structural stabilization of the Al-free NiCr-RMMO. In the absence of Al as a structural support, the NiCr-RMMO undergoes severe aggregation and phase segregation, which may promote extensive reconstruction of the Cr-containing phase under the long-term reaction conditions. In comparison, the Al-containing NiAl-RMMO and NiCrAl-RMMO retain their overall phase structures much more effectively, highlighting the important role of Al in stabilizing the catalyst architecture during prolonged operation.

**Figure S37.** Post-stability TEM characterization of the RMMO catalysts after 100 h of NH_3_ decomposition: TEM and HAADF-STEM images with corresponding Ni particle-size distributions of (a–c) NiAl-RMMO, (d–f) NiCr-RMMO, and (g–i) NiCrAl-RMMO.

The morphological evolution of the RMMO catalysts after the 100 h stability test was further investigated by TEM and HAADF-STEM analysis. Consistent with the XRD results, NiCr-RMMO exhibited severe particle aggregation after prolonged NH_3_ decomposition, accompanied by a substantial increase in particle size. The average particle size of the spent NiCr-RMMO reached approximately 162.0 nm, indicating extensive sintering and structural coarsening during the long-term reaction.

In contrast, the Al-containing NiAl-RMMO and NiCrAl-RMMO catalysts retained much smaller particle sizes after 100 h of operation. Their average particle sizes were 15.56 and 15.43 nm, respectively, showing only limited particle growth compared with NiCr-RMMO. This pronounced difference indicates that the presence of Al provides effective structural stabilization and suppresses the aggregation and sintering of Ni-containing particles under prolonged high-temperature reaction conditions. In particular, NiCrAl-RMMO maintained a highly dispersed nanostructure comparable to that of NiAl-RMMO, supporting the important role of the Al-containing matrix in preserving the structural integrity of the catalyst during long-term ammonia decomposition.

**Figure S38.** Post-stability HAADF-STEM images and corresponding EDS elemental maps of (a) NiAl-RMMO, (b) NiCr-RMMO, and (c) NiCrAl-RMMO after 100 h of NH_3_ decomposition.

TEM–EDS mapping of the spent catalysts further confirmed the differences in structural stability after the 100 h test. NiCr-RMMO showed severe aggregation, with Ni concentrated in large localized domains. In contrast, NiAl-RMMO and NiCrAl-RMMO maintained relatively homogeneous Ni distributions throughout the Al-containing matrix. These results further support the role of Al in suppressing metal aggregation during long-term ammonia decomposition.

**Figure S39.** Post-stability characterization of the RMMO catalysts after the 100 h stability test: (a) Ni 2p XPS spectra, (b) XPS-derived surface N concentrations, and (c) BET specific surface areas of NiAl-RMMO, NiCr-RMMO, and NiCrAl-RMMO.

The surface chemical states and textural properties of the spent catalysts after the 100 h stability test are summarized in Figure S39. The metallic Ni 2p_3/2_ peaks of all catalysts remained at binding energies similar to those of the fresh samples and preserved the same relative trend, indicating that the metallic Ni state was largely maintained during long-term operation. Surface N analysis showed slightly higher N accumulation on NiAl-RMMO than on NiCrAl-RMMO, suggesting somewhat greater nitrogen adsorption on NiAl-RMMO. In contrast, NiCr-RMMO exhibited a markedly higher N concentration, consistent with the formation of crystalline CrN observed by XRD. The BET surface areas of the spent catalysts slightly decreased after the stability test but were generally retained, particularly for the Al-containing NiAl-RMMO and NiCrAl-RMMO catalysts.

**Table S3**. Performance comparison of recently reported Ni/Al_2_O_3_-based catalysts for NH_3_ decomposition at 500 °C.

| Catalyst | Year | WHSV  (mL g^−1^ h^−1^) | Catalyst amount (mg) | Preparation method | H_2_ production rate  (mmol g_cat_^−1^ min^−1^) | Activation energy  (kJ/mol) | TOF_NH3_  (s^−1^) | Ref. |
| --- | --- | --- | --- | --- | --- | --- | --- | --- |
| **NiCrAl-RMMO** | **2026** | **30,000** | **60** | **Hydrothermal** | **22.83** | **31.3** | **2.67** | **This work** |
| Ni–La/Al_2_O_3_ | 2015 | 6,000 | - | Impregnation | 4.35 | 81.2 | 0.88^b^ | [S7] |
| Ni/MgAl LDH | 2017 | 30,000 | 200 | Co-precipitation | 7.37 | 92.8 | 0.20^a^ | [S8] |
| NiMgAl LDH | 2017 | 30,000 | 100 | Co-precipitation | 13.06 | 74.6 | 0.24^b^ | [S9] |
| Ni/Zr-Al_2_O_3_ | 2018 | 7,500 | 400 | Impregnation | 3.52 | 39.3 | 0.35^b^ | [S10] |
| Ni/Ba–Al–O | 2020 | 6,000 | 300 | Impregnation | 3.62 | 76.5 | 0.72^b^ | [S11] |
| Ni/Al_0.8_Ce_0.2_ZrO_2_ | 2020 | 9,000 | 100 | Impregnation | 5.93 | 66.8 | 0.48^a^ | [S12] |
| Ni/CaAl LDH | 2021 | 6,000 | 100 | Co-precipitation | 5.69 | 44.2 | 0.95^c^ | [S13] |
| Ni/Al_2_O_3_ | 2021 | 24,000 | 100 | One-pot soft-template | 13.39 | 62.6 | -^d^ | [S14] |
| Ni–Ce–Al | 2022 | 6,000 | 100 | One-pot CADH | 5.02 | 72.7 | 0.61^b^ | [S15] |
| Ni/MgAl LDH | 2022 | 30,000 | 500 | Hydrothermal | 19.09 | 39.2 | 0.94^b^ | [S16] |
| NiCeO_x_/Al_2_O_3_ | 2023 | 30,000 | 50 | Deposition-precipitation | 17.24 | 89.0 | -^d^ | [S17] |
| Ni/Al_2_O_3_ | 2024 | 6,000 | 100 | One-pot CADH | 4.35 | 79.2 | 0.36^b^ | [S18] |
| La/NiAl LDH | 2025 | 30,000 | 100 | Solvothermal | 13.39 | 74.3 | 0.40^b^ | [S19] |
| Ni/Al_2_O_3_ | 2025 | 30,000 | 50 | impregnation | 18.42 | - | -^d^ | [S20] |

^a^ TOF directly reported in the original reference.

^b^ TOF values were recalculated using the experimentally measured Ni dispersion or exposed surface Ni amounts reported in the corresponding references.

^c^ For Ref. [S13], the exposed Ni amount was estimated from H_2_ chemisorption by assuming H/Ni_surface_=1(H_2_/Ni_surface_=0.5).

^d^ Not calculated owing to the absence of experimentally determined Ni dispersion or exposed Ni amount.

All reactions were performed at atmospheric pressure unless otherwise noted.

**Figure S40.** In situ DRIFTS spectra of CO adsorption on (a) NiAl-RMMO, (b) NiCr-RMMO, and (c) NiCrAl-RMMO collected as a function of adsorption time.

**Figure S41.** Experimental procedure for in situ DRIFTS analysis, showing the temperature program and gas-feeding sequence for NH_3_ adsorption and desorption.

**Figure S42.** N_2_-TPD profiles of RMMO nanostructures at different ramping rates.

**Figure S43.** Comparison of the total adsorption amounts of (a) NH_3_, (b) CO_2_, and (c) H_2_ on the RMMO catalysts, as determined by TPD.

**Table S4**. Gas adsorption capacities of different RMMO nanostructures determined by TPD.

|  | NH_3_ adsorption amount  (mmol g^−1^) | CO_2_ adsorption amount  (mmol g^−1^) | H_2_ adsorption amount  (mmol g^−1^) | N_2_ adsorption amount  (mmol g^−1^) |
| --- | --- | --- | --- | --- |
| NiAl-RMMO | 0.363 | 1.486 | 0.340 | 1.770 |
| NiCr-RMMO | 0.310 | 0.621 | 0.130 | 0.784 |
| NiCrAl-RMMO | 0.451 | 1.450 | 0.166 | 2.623 |

**Figure S44.** NH_3_ reaction-order plots for NiAl-RMMO and NiCrAl-RMMO, obtained from the linear relationship between ln(r_NH3_) and ln(P_NH3_).

**Figure S45.** DFT-calculated adsorption energetics of N, N_2_ and NH_3_ on Ni and Ni–Cr cluster models. (a,b) Optimized adsorption configurations of atomic N on the Ni cluster and Ni–Cr cluster, respectively. (c) Calculated nitrogen adsorption energies on the two cluster models. (d,e) Optimized adsorption configurations of NH_3_ on the Ni cluster and Ni–Cr cluster, respectively. (f) Calculated NH_3_ adsorption energies on the two cluster models. (g,h) Optimized adsorption configurations of N_2_ on the Ni cluster and Ni–Cr cluster, respectively. (i) Calculated N_2_ adsorption energies on the two cluster models.

**References**

[S1] G. Kresse, *Journal of Non-Crystalline Solids* 1995, 192-193, 222. https://doi.org/10.1016/0022-3093(95)00355-X

[S2] G. Kresse, J. Hafner, *Journal of non-crystalline solids* 1993, 156, 956.

[S3] P. E. Blöchl, *Physical review B* 1994, 50, 17953.

[S4] X. Duan, J. Ji, G. Qian, C. Fan, Y. Zhu, X. Zhou, D. Chen, W. Yuan, *Journal of Molecular Catalysis A: Chemical* 2012, 357, 81.

[S5] J. Gu, J. Wang, J. Leszczynski, *ACS omega* 2018, 3, 1881.

[S6] S. Manna, Y. Wang, A. Hernandez, P. Lile, S. Liu, T. Mueller, *Scientific Data* 2023, 10, 308.

[S7] K. Okura, T. Okanishi, H. Muroyama, T. Matsui, K. Eguchi, *Applied Catalysis A: General* 2015, 505, 77. https://doi.org/10.1016/j.apcata.2015.07.020

[S8] K. Sato, N. Abe, T. Kawagoe, S.-i. Miyahara, K. Honda, K. Nagaoka, *International Journal of Hydrogen Energy* 2017, 42, 6610. https://doi.org/10.1016/j.ijhydene.2016.11.150

[S9] Q. Su, L. Gu, Y. Yao, J. Zhao, W. Ji, W. Ding, C.-T. Au, *Applied Catalysis B: Environmental* 2017, 201, 451. https://doi.org/10.1016/j.apcatb.2016.08.051

[S10] S. Henpraserttae, S. Charojrochkul, W. Klysubun, L. Lawtrakul, P. Toochinda, *Catalysis Letters* 2018, 148, 1775. https://doi.org/10.1007/s10562-018-2381-9

[S11] Y. Im, H. Muroyama, T. Matsui, K. Eguchi, *International Journal of Hydrogen Energy* 2020, 45, 26979. https://doi.org/10.1016/j.ijhydene.2020.07.014

[S12] D. Sima, H. Wu, K. Tian, S. Xie, J. J. Foo, S. Li, D. Wang, Y. Ye, Z. Zheng, Y.-Q. Liu, *International Journal of Hydrogen Energy* 2020, 45, 9342. https://doi.org/10.1016/j.ijhydene.2020.01.209

[S13] L. Deng, H. Lin, X. Liu, J. Xu, Z. Zhou, M. Xu, *International Journal of Hydrogen Energy* 2021, 46, 38351. https://doi.org/10.1016/j.ijhydene.2021.09.071

[S14] Y. Gu, Y. Ma, Z. Long, S. Zhao, Y. Wang, W. Zhang, *International Journal of Hydrogen Energy* 2021, 46, 4045. https://doi.org/10.1016/j.ijhydene.2020.11.003

[S15] Q. C. Do, Y. Kim, T. A. Le, G. J. Kim, J.-R. Kim, T.-W. Kim, Y.-J. Lee, H.-J. Chae, *Applied Catalysis B: Environment and Energy* 2022, 307. https://doi.org/10.1016/j.apcatb.2022.121167

[S16] Y. Qiu, E. Fu, F. Gong, R. Xiao, *International Journal of Hydrogen Energy* 2022, 47, 5044. https://doi.org/10.1016/j.ijhydene.2021.11.117

[S17] L.-L. Zhou, X.-P. Fu, Y.-S. Xu, W.-W. Wang, C.-J. Jia, *ACS Applied Nano Materials* 2023, 6, 2952. https://doi.org/10.1021/acsanm.2c05345

[S18] Q. C. Do, Y. Kim, G. J. Kim, Y. Im, T. A. Le, G. H. Kim, K. C. Ko, H.-J. Chae, *Chemical Engineering Journal* 2024, 501. https://doi.org/10.1016/j.cej.2024.157605

[S19] Z. Li, G.-H. Chen, X.-C. Gong, J.-J. Yao, J.-K. Guo, L. Chen, C. Peng, X.-Y. Huang, Q. Hu, J. Xiong, S.-F. Yin, *Chemical Engineering Science* 2025, 304. https://doi.org/10.1016/j.ces.2024.121000

[S20] P. K. Yadav, S. Gunge, A. S. Russel, G. Deo, *Applied Catalysis B: Environment and Energy* 2025, 371. https://doi.org/10.1016/j.apcatb.2025.125205
